# A food-sensitive olfactory circuit drives anticipatory satiety

**DOI:** 10.1038/s42255-025-01301-1

**Published:** 2025-06-11

**Authors:** Janice Bulk, Joscha N. Schmehr, Tobias Ackels, Rui de Oliveira Beleza, André Carvalho, Ayden Gouveia, Lionel Rigoux, Vincent Hellier, Anna Lena Cremer, Heiko Backes, Andreas Schaefer, Sophie M. Steculorum

**Affiliations:** 1 Max Planck Institute for Metabolism Research, Max Planck Research Group Neurocircuit Wiring and Function, Cologne, Germany; 2 Excellence Cluster on Cellular Stress Responses in Aging-Associated Diseases (CECAD), University of Cologne, Cologne, Germany; 3 Sensory Circuits and Neurotechnology Lab, The Francis Crick Institute, London, UK; 4 University of Bonn, Faculty of Medicine, Institute for Experimental Epileptology and Cognition Research (IEECR), Bonn, Germany; 5 Max Planck Institute for Metabolism Research, Translational Neurocircuitry Group, Cologne, Germany; 6 Max Planck Institute for Metabolism Research, Multimodal Imaging Group, Cologne, Germany; 7 German Center of Diabetes Research (DZD), Neuherberg, Germany

**Keywords:** Feeding behaviour, Obesity, Metabolism

## Abstract

Food sensory perception has emerged as a potent regulator of specialized feeding circuits; yet, the consequences on feeding behaviour and the underlying neuronal basis remain poorly understood. Here, we reveal a sensory pathway that co-ordinately integrates food odours to control forthcoming nutrient intake in male mice. Unbiased whole-brain mapping of food odour-induced brain activity revealed a potent activation of the medial septum (MS), where food odours selectively activate MS glutamatergic neurons (MS^VGLUT2^). Activity dynamics of MS^VGLUT2^ neurons uncovered a biphasic modulation of their neuronal activity with a transient activation after detection of food odours and a long-lasting inhibition following food ingestion, independent of the caloric value and identity of the food. MS^VGLUT2^ neurons receive direct projections from the olfactory bulb (OB) and acute optogenetic stimulation of OB→MS projections selectively before food ingestion decreased feeding in lean mice. However, acute OB→MS optogenetic stimulation in diet-induced obese mice failed to reduce feeding, suggesting the involvement of this pathway in calorie-rich diet-induced hyperphagia and obesity development. Altogether, our study uncovered a sensory circuit by which the organism integrates olfactory food cues to prime satiety at the outset of a meal.

## Abstract (web summary)

The authors describe a sensory circuit involving the medial septum (MS), where MS glutamatergic neurons integrate food odours to prime satiety and regulate nutrient intake.

## Main

Tight regulation of feeding behaviour requires the co-ordinated integration of homeostatic signals aligned with the detection of environmental cues^1^. The perception of food sensory cues before a meal triggers cephalic phase responses, which are anticipatory physiological responses preparing an animal to respond optimally to the upcoming nutrients^2–5^. A seminal example of these cephalic phase responses is Pavlov’s discovery revealing that exposure to food cues before food ingestion increases salivary, gastric and pancreatic secretions^6,7^. Recent studies further demonstrated that food sensory cues, that is, sight, smell and learned predictive cues, also exert potent effects on peripheral metabolism, including hepatic homeostasis, brown adipose tissue thermogenesis and lipolysis^8–11^. Although we have begun to comprehend the pivotal role of food sensory cues in whole-body metabolism, their role in feeding regulation itself, the contributory role of distinct sensory modalities and the underlying neurocircuits remain poorly understood.

Exposure to food sensory cues rapidly reverses the neuronal activity state of key feeding-regulatory neurons^12–16^. Previous studies revealed that food–predictive cues can inhibit neurons promoting the drive to eat and activate those decreasing it, suggesting that sensory detection of food may trigger an anorexigenic tone. In detail, food cues acutely dampen the activity of orexigenic neurons, that is, hunger neurons expressing agouti-related peptide (AgRP), and increase the activity of anorexigenic neurons, that is, proopiomelanocortin (POMC)-expressing and glucagon-like peptide-1 receptor (GLP-1R)-expressing neurons in the paraventricular nucleus of the hypothalamus (PVH)^12,13,15,16^. However, post-ingestive signals are needed for sustained changes in neuronal activity^17–20^. Although the physiological and behavioural relevance of the sensory regulation of feeding-regulatory neurons remains unclear, it has been proposed to serve as an anticipatory mechanism allowing the animal to prime satiation and satiety processes^21^. Furthermore, the sensory regulation of these feeding-related neurons is blunted in obesity^22,23^, suggesting a role of their sensory regulation in the establishment or progression of obesity and associated metabolic and behavioural outcomes. The sensory regulation of neuronal activity has been a paradigm-shifting discovery challenging the long-lasting assumption that the activity of key feeding-regulatory neurons is mainly controlled by post-ingestive and adiposity signals. Still, the behavioural relevance of these processes and their contribution to obesity is yet to be elucidated.

Here, we aimed to investigate how an organism integrates anticipatory food cues before a meal and the consequences on feeding behaviour in health and obesity. Because food sensory regulation of neuronal activity is triggered by hidden food or food odours alone^13,24^, we selectively focused on olfactory circuits and the contributory role of food odours. Collectively, we uncovered an olfactory pathway able to integrate food odours and prime satiety at the outset of a meal.

## Results

### Unbiased whole-brain mapping of food odour-sensitive regions

**Fig. 1:**
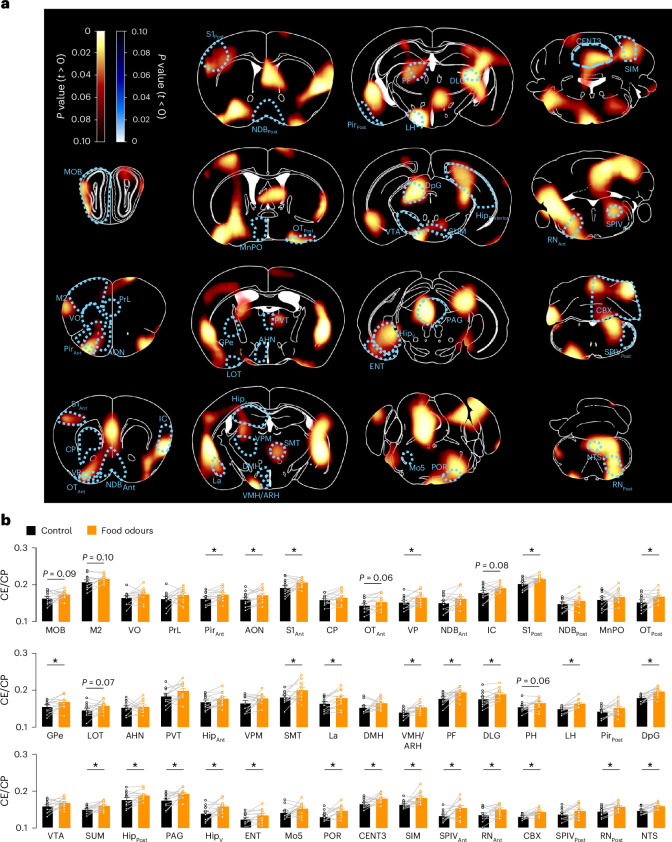
Functional mapping of brain regions activated by food odours. **a**, Voxel-based analyses of the ratio of tissue and plasma glucose concentrations (CE/CP) based on ^18^F-FDG kinetics in brains of fasted C57BL/6N mice not exposed or exposed to food odours (NCD). The yellow–red and blue–white scales represent the magnitude of *P* values from a voxel-wise paired *t*-test indicating food odour-induced activated regions (yellow–red) or inhibited regions (blue–white; paired two-tailed Student’s *t*-test; *n* = 12 mice). Atlas-based anatomical brain regions analysed are indicated by dashed blue lines. **b**, Quantification of ^18^FDG metabolism of regions depicted in **a**. Data are represented as the mean ± s.e.m. *P ≤ 0.05 (dark bars indicate mice not exposed to food odours; orange bars indicate mice exposed to food odours). AHN, anterior hypothalamic nuclei; AON, anterior olfactory nuclei; ARH, arcuate nucleus of the hypothalamus; CBX, cerebellar cortex; CENT3, cerebellum lobule III; CP, caudoputamen; DLG, dorsal lateral geniculate nucleus; DMH, dorsomedial nucleus of the hypothalamus; DpG, deep grey layer of the superior colliculus; ENT, entorhinal cortex; GPe, globus pallidus; Hip_Ant_, anterior section of hippocampus; Hip_Post_, posterior section of hippocampus; Hip_V_, ventral section of hippocampus; IC, insular cortex; La, lateral amygdaloid nucleus; LH, lateral hypothalamus; M2, secondary motor cortex; MnPO, median preoptic nucleus; Mo5, trigeminal motor nucleus; MOB, main olfactory bulb; MPOA, medial preoptic area; NDB_Ant_, anterior section of diagonal band nucleus; NDB_Post_, posterior section of diagonal band nucleus; NTS, nucleus of the solitary tract; OT_Ant_, anterior section of olfactory tubercle; OT_Post_, posterior section of olfactory tubercle; PAG, periaqueductal grey area; PF, prefrontal cortex; PH, posterior hypothalamic area; Pir_Ant_, anterior section of piriform cortex; Pir_Post_, posterior section of piriform cortex; POR, Superior olivary complex; PrL, prelimbic cortex; PVT, paraventricular thalamic nucleus; RN_Ant_, anterior section of the reticular nucleus; RN_Post_, posterior section of the reticular nucleus; S1_Ant_, anterior section of primary somatosensory cortex; S1_Post_, posterior section of primary somatosensory cortex; SIM, simple lobule; SMT, submedial nucleus of the thalamus; SPIV_Ant_, anterior section of spinal vestibular nucleus; SPIV_Post_, posterior section of spinal vestibular nucleus; SUM, supramammillary nucleus; VMH, ventromedial nucleus of the hypothalamus; VO, ventral orbital cortex; VP, ventral pallidum; VPM, ventral posteromedial nucleus of the thalamus; VTA, ventral tegmental area. Source data

**Extended Data Fig. 1:**
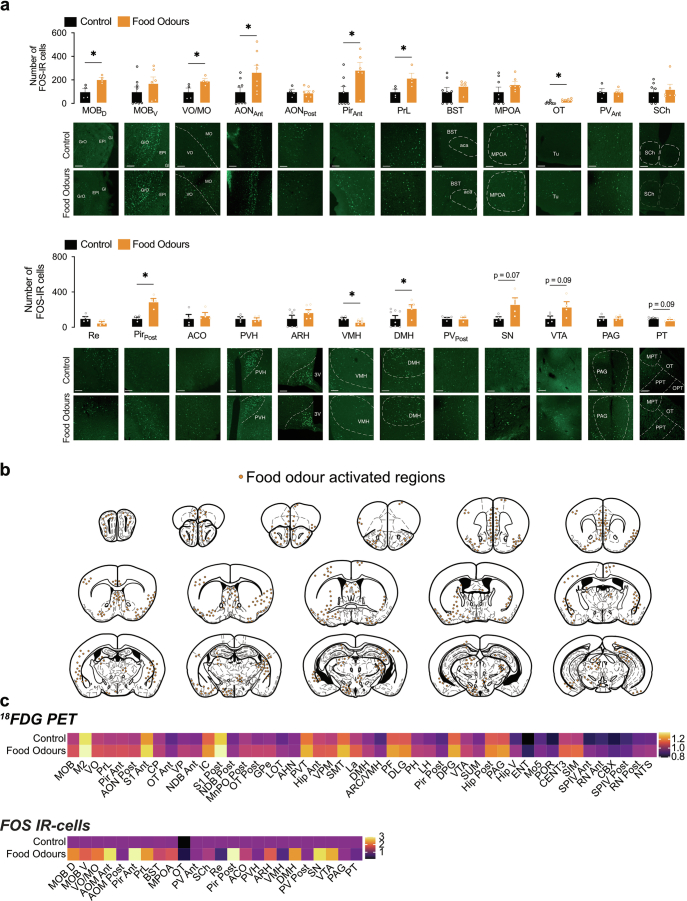
Unbiased whole-brain mapping of food odour activation. **a)** Representative images and quantification comparison of food odour-induced (normal chow diet, NCD) FOS immunoreactivity (IR) in fasted C57BL/6 N mice [unpaired two-tailed Student’s t-test; n = 4-8 mice]. Scale bar: 100 µm. **b)** Schematic representation of brain regions showing a food odour-induced (NCD) increased glucose uptake and FOS immunoreactivity. **c)** Heatmap of ^18^FDG and number of FOS-positive cells per region normalized to overall average ^18^FDG and cell number in mice exposed to no odours. Data are represented as mean ± SEM. *p ≤ 0.05, **p ≤ 0.01, ***p ≤ 0.001 and, ****p ≤ 0.0001. ACO, anterior cortical amygdaloid nucleus; AON_Ant_, anteromedial part of the accessory olfactory nucleus; AON_Post_, posteromedial part of the accessory olfactory nucleus; ARH, arcuate nucleus of the hypothalamus; BST, bed nucleus of the stria terminals; DMH, dorsomedial nucleus of the hypothalamus; MOB_D_, dorsal main olfactory bulb; MOB_V_, ventral main olfactory bulb; MO, medial orbital cortex; MPOA, medial preoptic area; OPT, olivary pretectal nucleus; OT, olfactory tubercle; PAG, periaqueductal grey area; Pir_Ant_, anterior section of the piriform cortex; Pir_Post_, posterior section of the piriform cortex; PrL, prelimbic cortex; PV_Ant_, anterior section of paraventricular thalamic nucleus; PV_Post_, posterior section of paraventricular thalamic nucleus; PVH, paraventricular hypothalamic nucleus; Re, reuniens thalamic nucleus; SCh, suprachiasmatic nucleus; SN, substantia nigra; VMH, ventromedial nucleus of the hypothalamus; VO, ventral orbital cortex; VTA, ventral tegmental area. Source data

To identify candidate circuits integrating olfactory food cues, we performed an unbiased mapping of neuronal activation following food odour exposure. We first used ^18^F-fluorodeoxyglucose (^18^FDG) positron emission tomography (PET) imaging to quantify glucose transport in distinct brain areas as a proxy for in vivo neuronal activation. Olfactory exposure to a normal chow diet (NCD) in overnight-fasted C57BL/6N mice induced an increase of glucose transport in canonical regions involved in odour processing such as the anterior olfactory nuclei, piriform cortex, olfactory tubercle and entorhinal cortex (Fig. 1a,b and Extended Data Fig. 1b,c). Beyond the activation of olfactory-related regions, olfactory food exposure also increased glucose transport in hypothalamic nuclei such as the arcuate nucleus of the hypothalamus, the ventromedial nucleus of the hypothalamus and the lateral hypothalamus (Fig. 1a,b and Extended Data Fig. 1b,c). Glucose uptake also significantly rose in other brain areas regulating feeding such as the nucleus tractus solitary and the periaqueductal grey (Fig. 1a,b and Extended Data Fig. 1b,c). We complemented the in vivo ^18^FDG-PET imaging with unbiased whole-brain mapping of neuronal activation in response to olfactory food exposure using immunostaining for the immediate early response gene *FOS* (Extended Data Fig. 1a–c). Food odours increased the number of FOS immunoreactive cells in analogous regions identified by ^18^FDG-PET-based experiments, including olfactory and hypothalamic regions (Extended Data Fig. 1a–c). Collectively, these findings uncover a specific signature of neuronal activation in response to food odour exposure.

### Food odours potently activate the MS

**Fig. 2:**
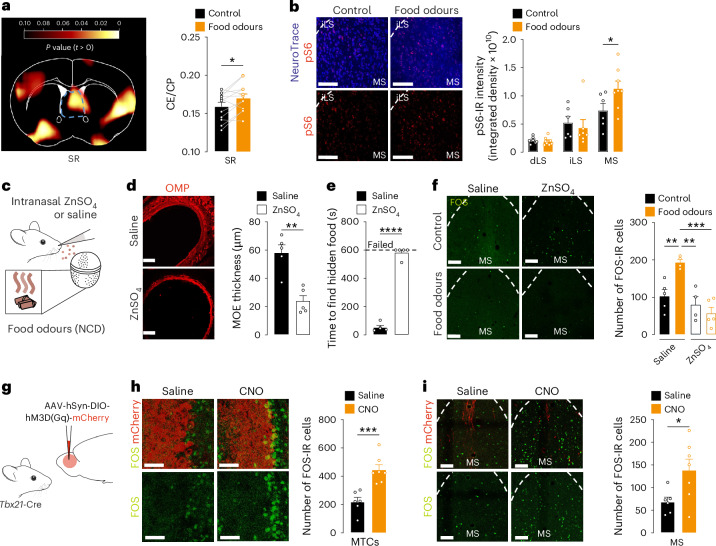
Food odours activate the MS. **a**, Voxel-based analyses of the ratio of tissue and plasma glucose concentrations (CE/CP) in the septal region (SR; blue box) in mice not exposed or exposed to food odours. The yellow–red scale represents the magnitude of *P* values. Results are plotted as the means of *P* values determined by paired Student’s *t*-test between control and food odour exposure in the SR (paired two-tailed Student’s *t*-test; *n* = 12 mice; *P* = 0.018). **b**, Representative images of the MS and quantification comparison of the dorsal lateral septum (dLS), intermediate lateral septum (iLS) and MS of food odour-induced phosphorylated S6 (pS6) immunoreactivity (IR; unpaired Student’s *t*-test; *n* = 6 mice for control, *n* = 8 mice for food odours; *P* = 0.049; pS6, red; NeuroTrace, blue). Scale bars, 100 µm. **c**, Schematic of ZnSO_4_-induced anosmia. **d**, Representative images and quantification comparison of the thickness of the MOE (immunoreactive for the olfactory marker protein, OMP, red) in ZnSO_4_-treated or saline-treated mice (unpaired two-tailed Student’s *t*-test; *n* = 5 mice; *P* = 0.001). Scale bars, 250 µm. **e**, Food finding test in ZnSO_4_-treated or saline-treated mice. ‘Failed’ refers to unsuccessful retrieval of the cookie within 10 min (unpaired two-tailed Student’s *t*-test; *n* = 5 mice; *P* > 0.0001). **f**, Representative images and quantification comparison of food odour-induced FOS immunoreactivity (green) in ZnSO_4_-treated or saline-treated mice (mixed-effects multiple-comparison analysis; *n* = 4 mice for saline control, ZnSO_4_ food odours; *n* = 5 for saline food odours, ZnSO_4_ control; *P* = 0.038, *P* = 0.02, *P* = 0.0097). Scale bars, 100 µm. **g**, Paradigm for chemogenetic activation of MTCs. **h**, Representative images and quantification comparison of CNO-induced or saline control-induced FOS immunoreactivity (green) in MTCs (mCherry-positive, red) in the OB (unpaired two-tailed Student’s *t*-test; *n* = 6 mice for saline, *n* = 7 mice for CNO injection; *P* = 0.0007). Scale bars, 50 µm. **i**, Representative images and quantification of CNO-induced FOS immunoreactivity in the MS as compared to saline-injected mice (unpaired two-tailed Student’s *t*-test; *n* = 6 mice for saline, *n* = 7 mice for CNO injection; *P* = 0.032; FOS, green; mCherry, red). Scale bars, 100 µm. Data are represented as the mean ± s.e.m. *P ≤ 0.05, **P ≤ 0.01, ***P ≤ 0.001, ****P ≤ 0.0001. Mouse images in **c** and **g** adapted from SciDraw under a Creative Commons license CC BY 4.0. Source data

**Extended Data Fig. 2:**
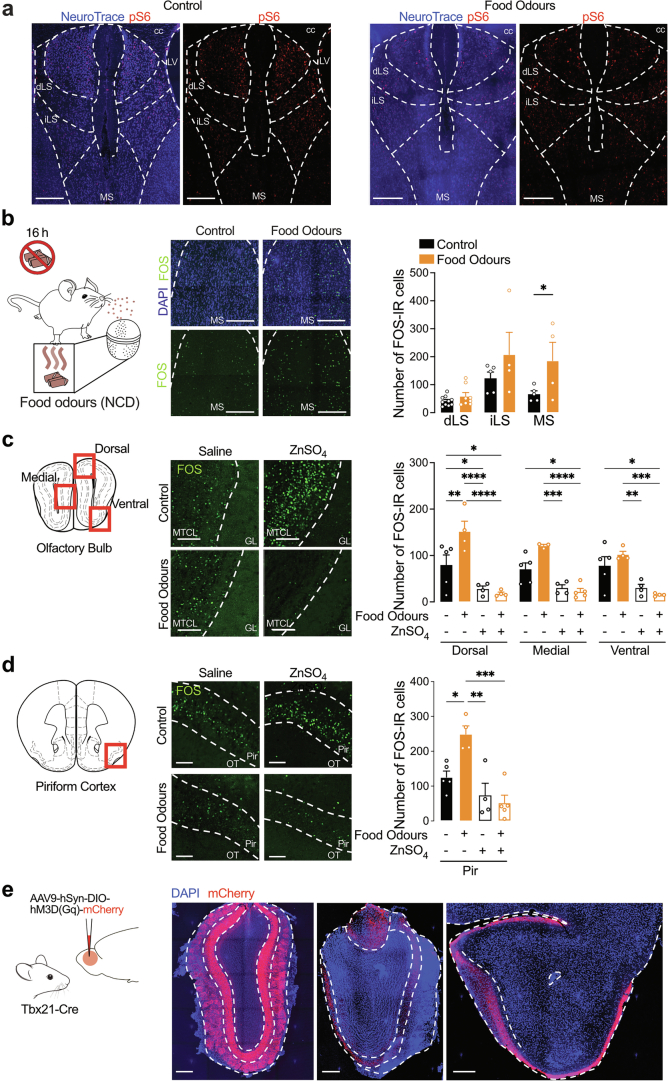
The food odour-induced activation is blunted in anosmic mice. **a)** Representative images of food odour-induced (normal chow diet, NCD) pS6 immunoreactivity (pS6, red; NeuroTrace, blue) in the dorsal lateral septum (dLS), intermediate lateral septum (iLS) and medial septum (MS). Scale bar: 250 µm. **b)** Representative images of the MS and quantification comparison of food odour-induced (normal chow diet, NCD) FOS immunoreactivity (IR) (FOS, green) of the dorsal lateral septum (dLS), intermediate lateral septum (iLS) and medial septum (MS) in C57BL/6 N mice after an 16 h overnight fast [mixed-effect multiple comparisons analysis; n = 4 mice for saline control, ZnSO_4_ food odours; n = 5 for saline food odours, ZnSO_4_ control]. Scale bar: 100 µm. **c**-**d)** Representative images and quantification comparison of food odour-induced (NCD) FOS immunoreactivity (FOS, green; Neurotrace, blue) after intranasal ZnSO_4_ or saline administration in **c)** the dorsal olfactory bulb, medial olfactory bulb and ventral olfactory bulb [mixed-effect multiple comparisons analysis; n = 4 mice for saline control, ZnSO_4_ food odours; n = 5 for saline food odours, ZnSO_4_ control] and **d)** the piriform cortex (Pir) [mixed-effect multiple comparisons analysis; n = 4 mice for saline control, ZnSO_4_ food odours; n = 5 for saline food odours, ZnSO_4_ control] Scale bar: 100 µm. **e)** Representative images of the viral spread throughput the olfactory bulb after AAV9-hSyn-DIO-hM3DGq-mCherry (mCherry, red; DAPI, blue) injection into the olfactory bulb. Scale bar: 250 µm. Data are represented as mean ± SEM. *p ≤ 0.05, **p ≤ 0.01, ***p ≤ 0.001 and, ****p ≤ 0.0001. dLS, dorsal lateral septum; iLS, intermediate lateral septum; MS, medial septum; GL, glomerular layer; MTCL, mitral and tufted cell layer; Pir, piriform cortex; OT, olfactory tubercle. Mouse image in **e** adapted from SciDraw under a Creative Commons license CC BY 4.0. Panel **b** created using BioRender.com. Source data

Alongside the aforementioned changes in activity across olfactory and feeding-regulatory regions (Fig. 1 and Extended Data Fig. 1), we observed an increase in glucose transport in the septal region following food odour exposure (Fig. 2a). The septal region recently received interest for its feeding-regulatory effects^25–33^. As its involvement in the sensory detection of food remains unexplored, we decided to selectively focus on this region. Because the septal region is a large region conventionally divided into subregions, we used immunostaining to map and quantify the exact location of food odour-activated cells (Fig. 2b). Mapping of food odour-activated cells using phosphorylation of the ribosomal protein S6 (pS6) as a proxy for neuronal activation revealed that food odours did not evoke changes in pS6 immunoreactivity in the dorsal lateral septum or in the intermediate lateral septum (Fig. 2b and Extended Data Fig. 2a). Conversely, pS6 immunoreactivity (Fig. 2b) and number of FOS-positive cells (Extended Data Fig. 2b) increased in response to food odours in the MS. Hence, food odours selectively and exclusively activate the MS within the septal region.

To assess the direct contribution of the olfactory system in the food odour-induced activation in the MS, we reiterated the mapping of neuronal activation in response to food odours in anosmic mice. We used a chemical model of anosmia induced by intranasal administration of zinc sulfate (ZnSO_4_; Fig. 2c), which is a compound routinely used to degenerate the main olfactory epithelium (MOE)^34–37^. Accordingly, ZnSO_4_ administration diminished the MOE thickness compared to control mice, as revealed by a decreased thickness of the layer of cells expressing the olfactory marker protein, that is, a marker of olfactory sensory neurons (Fig. 2d). ZnSO_4_-induced anosmia was also confirmed by a behavioural test in which ZnSO_4_-treated mice failed to perform in a food finding test (Fig. 2e). Further, the food odour-induced FOS activation in the OB and the piriform cortex observed in control mice was blunted in ZnSO_4_-treated mice (Extended Data Fig. 2c,d). While food odour exposure increased the number of FOS immunoreactive cells in the MS in control saline-treated mice, no increase was observed in the MS in anosmic mice (Fig. 2f), demonstrating that olfaction is required for the food odour-induced activation of the MS.

To further corroborate a link between the activation of the olfactory system and the MS, we next used chemogenetics to artificially activate the excitatory mitral and tufted cells (MTCs), that is, glutamatergic projection neurons of the OB^38,39^. MTCs represent the first level of olfactory processing in the central nervous system, which, following odour detection, receive excitatory input from olfactory sensory neurons and convey signals to various brain regions^38,39^. To selectively target MTCs, we used mice expressing Cre recombinase under the *Tbx21* promoter that is restricted to MTCs in the central nervous system^40^. We delivered an adeno-associated virus (AAV)-hSyn-DIO-hM3DGq-mCherry bilaterally into the OB allowing for Cre-dependent expression of hM3DGq, that is, a stimulatory designer receptor exclusively activated by designer drugs (DREADD; Fig. 2g). Of note, we used the AAV9 serotype, which offers large diffusion properties in the brain parenchyma and allows the AAV to diffuse through the entire OB ventro-dorsal axis (Extended Data Fig. 2e). Bilateral AAV delivery in the OB yielded successful expression of hM3DGq in MTCs as revealed by the presence of the mCherry reporter in MTC layers (Fig. 2h and Extended Data Fig. 2e). The number of FOS immunoreactive MTCs increased twofold following clozapine-*N*-oxide (CNO) administration in mice expressing hM3DGq in MTCs (*Tbx21*^hM3DGq^ mice) as compared to saline-injected control mice (Fig. 2h). In addition, the number of FOS immunoreactive cells also increased in the MS following chemogenetic activation of MTCs (Fig. 2i), further demonstrating the existence of a link between the OB and the MS.

### Food odours selectively activate MS^VGLUT2^ neurons

**Fig. 3:**
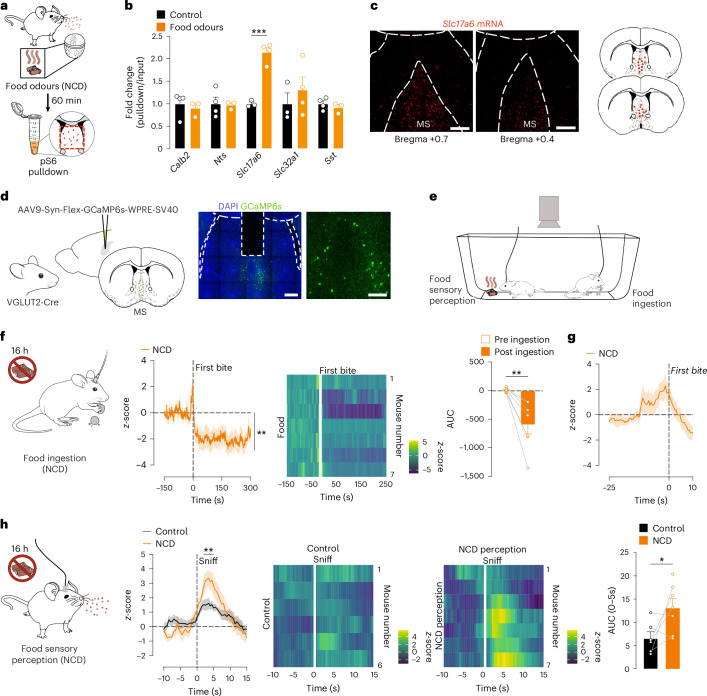
Sensory food perception activates MS^VGLUT2^ neurons. **a**, Paradigm of food odour exposure (NCD) and subsequent phosphorylated ribosome pulldown of the SR in fasted C57BL/6N mice. **b**, Quantification comparison of food odour-induced mRNA expression of neurons expressing *Calb2*, *Nts*, *Slc17a6*, *Slc32a1* and *Sst* as the fold change between pulldown and input sample (two-way analysis of variance (ANOVA); *n* = 34 pooled groups of five mice each; *P* = 0.0001). **c**, Representative images of *Slc17a6* (Vglut2) expression in the SR. Scale bars, 100 µm. **d**, Schematic of AAV-based expression of the calcium sensor GCaMP6s in MS^VGLUT2^ neurons and representative images of the fibre placement in VGLUT2-Cre mice (GCaMP6s, green; DAPI, blue). Scale bars, 100 µm. **e**, Paradigm for recording the activity dynamics of MS^VGLUT2^ neurons during food perception and food ingestion in freely behaving mice. **f**, MS^VGLUT2^ calcium dynamics and area under the curve (AUC) aligned to the onset of food consumption (NCD) in overnight-fasted mice; statistics indicate a significant difference between calcium signal before and after first bite (time curve: two-way ANOVA; *n* = 7; AUC: paired two-tailed Student’s *t*-test; *n* = 7 mice; *P* = 0.0068). **g**, MS^VGLUT2^ calcium dynamics before food ingestion (*n* = 7 mice). **h**, Calcium dynamics of MS^VGLUT2^ neurons and AUC aligned to the first olfactory exploration (that is, sniff) when perceiving food (NCD) or a non-edible familiar object (empty teaball; control) in overnight-fasted mice; line on top indicates time points with a significant difference in calcium signal between control and NCD conditions (time curve: paired two-way ANOVA, Dunnett’s post hoc; *n* = 6 mice for control, *n* = 7 mice for NCD perception; AUC: unpaired two-tailed Student’s *t*-test; *n* = 6 mice for control, *n* = 7 mice for NCD perception; *P* = 0.024). Data are represented as the mean ± s.e.m. *P ≤ 0.05, **P ≤ 0.01, ***P ≤ 0.001, ****P ≤ 0.0001. Mouse images in **d**–**f** and Eppendorf image in **a** adapted from SciDraw under a Creative Commons license CC BY 4.0. Panels **a** and **h** created using BioRender.com. Source data

To elucidate the molecular identity of food odour-sensitive neurons in the MS, we used a phosphorylated ribosome capture technique based on pS6 immunoprecipitation (pS6-RiboTrap; Fig. 3a)^41^. pS6-RiboTrap experiments were performed on septal region homogenates following olfactory food exposure to assess gene enrichment of molecular markers of known neuronal subpopulations residing in this region. Food odour exposure increased the gene expression of a vesicular glutamate transporter (*Slc17a6*, that is, VGLUT2) by twofold as compared to control odours without affecting the gene expression of the vesicular GABA transporter (*Slc32a1*, that is, VGAT), calretinin (*Calb2*), neurotensin (*Nts*) and somatostatin (*Sst*; Fig. 3b). Further, RNAScope-based mapping of *Slc17a6*-expressing neurons in the septal region revealed that *Slc17a6* neurons largely reside in the MS^42^ (Fig. 3c), in line with the anatomical location of food odour-responsive neurons in the MS (Fig. 2b). Thus, these results indicate that MS^VGLUT2^ neurons are selectively activated by food odours.

### Biphasic activity of MS^VGLUT2^ neurons during feeding

Having identified that food odours selectively activate MS^VGLUT2^ neurons, we next aimed to investigate the activity dynamics of these neurons in response to food sensory perception and ingestion in vivo. To measure MS^VGLUT2^ neuronal activity, we performed fibre photometry calcium recording in VGLUT2-Cre mice following Cre-dependent AAV-based expression of the genetically encoded calcium indicator GCaMP6s in MS^VGLUT2^ neurons (Fig. 3d). We first recorded MS^VGLUT2^ neuronal activity during food ingestion in a fasting–refeeding paradigm (Fig. 3e,f). As previously described by Xu et al.^27^, we observed that MS^VGLUT2^ neuronal activity rapidly decreased following food ingestion (Fig. 3f; MS^VGLUT2^ neuronal activity aligned to the first bite of a NCD pellet). Noticeably, this rapid drop in MS^VGLUT2^ neuronal activity was preceded by a fast, acute and substantial increase in activity (Fig. 3g; MS^VGLUT2^ neuronal activity before the aligned first bite of a NCD pellet). Since this peak in neuronal activity occurred shortly before the first bite, we next measured MS^VGLUT2^ neuronal activity during the sensory investigation of the food pellet, that is, smell and sight. Food sensory perception in overnight-fasted mice enhanced MS^VGLUT2^ neuronal activity compared with exposure to a non-edible familiar object (Fig. 3h; MS^VGLUT2^ neuronal activity aligned to the first sniff of the pellet or non-edible object). Collectively, the characterization of the activity dynamics of MS^VGLUT2^ neurons revealed a biphasic modulation of their neuronal activity with a transient activation following food sensory perception and a long-lasting inhibition following food ingestion.

**Extended Data Fig. 3:**
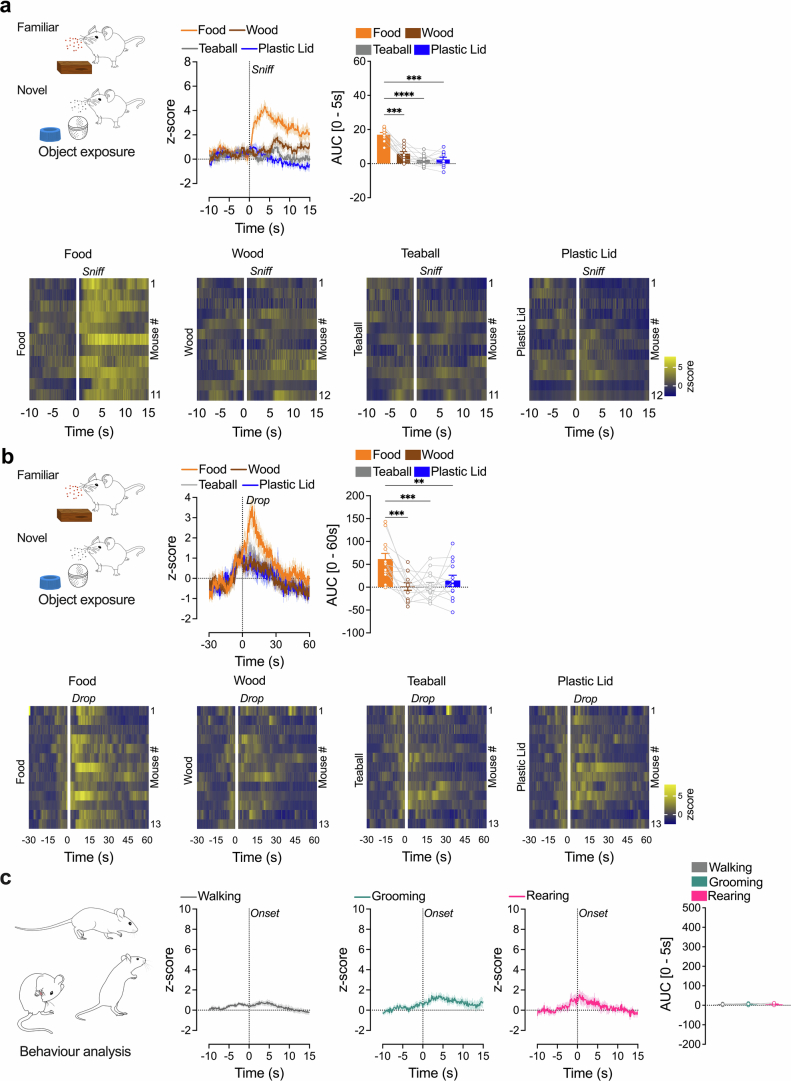
MS^VGLUT2^ neurons are not activated upon exposure to non-edible objects. **a)** MS^VGLUT2^ calcium dynamics and area under the curve (AUC) aligned to the onset of the first sniff when investigating a pellet of food (NCD), wooden stick (familiar object), a small plastic lid (small novel object, first exposure) or a metal teaball (big novel object, first exposure) [time curve: paired two-way ANOVA, Sidak post-hoc; n = 11 mice for sniffing food pellet and metal teaball, n = 12 mice for sniffing wooden stick and small plastic lid] [AUC: mixed effects multiple comparisons analysis; n = 11 mice for sniffing food pellet and metal teaball, n = 12 mice for sniffing wooden stick and small plastic lid; p = 0.0006, p = 0.0004, p < 0.0001]. **b)** MS^VGLUT2^ calcium dynamics and area under the curve (AUC) aligned to the drop of the non-edible objects (wooden stick, brown; metal teaball, grey; falcon lid, blue) or normal chow diet pellets (food, orange) into the experimental setup [time curve: paired two-way ANOVA, Sidak post-hoc; n = 13] [AUC: one-way ANOVA, Tukey post-hoc; n = 13 mice; p = 0.002, p = 0.037, p = 0.0002]. **c)** MS^VGLUT2^ calcium dynamics and AUC during walking, grooming and rearing behaviour during control exposure aligned to the onset of the behaviour [time curve: n = 6 mice for rearing, n = 13 mice for walking and grooming behaviour] [AUC: one-way ANOVA, Tukey post-hoc; n = 6-13 mice]. Data are represented as mean ± SEM. *p ≤ 0.05, **p ≤ 0.01, ***p ≤ 0.001 and, ****p ≤ 0.0001 Mouse images in **c** reproduced from SciDraw under a Creative Commons license CC BY 4.0. Panels **a** and **b** created using BioRender.com. Source data

To further assess the selectivity of the response towards food sensory cues, we performed additional control experiments using familiar (wooden stick) and novel (empty teaball and plastic lid) non-edible objects (Extended Data Fig. 3a,b). MS^VGLUT2^ neuronal activity remained unchanged following olfactory investigation (Extended Data Fig. 3a) and placing (Extended Data Fig. 3b) of non-edible familiar or unfamiliar objects. Instead, MS^VGLUT2^ neuronal activity increased specifically when an NCD pellet was introduced into the cage (Extended Data Fig. 3b), indicating that this activation is selectively driven by food sensory cues rather than generalized arousal or olfactory responses. In addition, since previous studies reported that MS^VGLUT2^ neurons residing in a more rostral part of the MS are activated at the onset of locomotion^43,44^, we further analysed the neuronal activity of the MS^VGLUT2^ neurons following locomotion (Extended Data Fig. 3c). Walking, grooming and rearing did not elicit changes in the neuronal activity of MS^VGLUT2^ neurons (Extended Data Fig. 3c). These experiments demonstrated that MS^VGLUT2^ neuronal activation in response to food sensory perception is not driven by changes in locomotion, or by generalized effects linked to arousal or olfactory investigation.

### Food odours potently activate MS^VGLUT2^ neurons in vivo

**Fig. 4:**
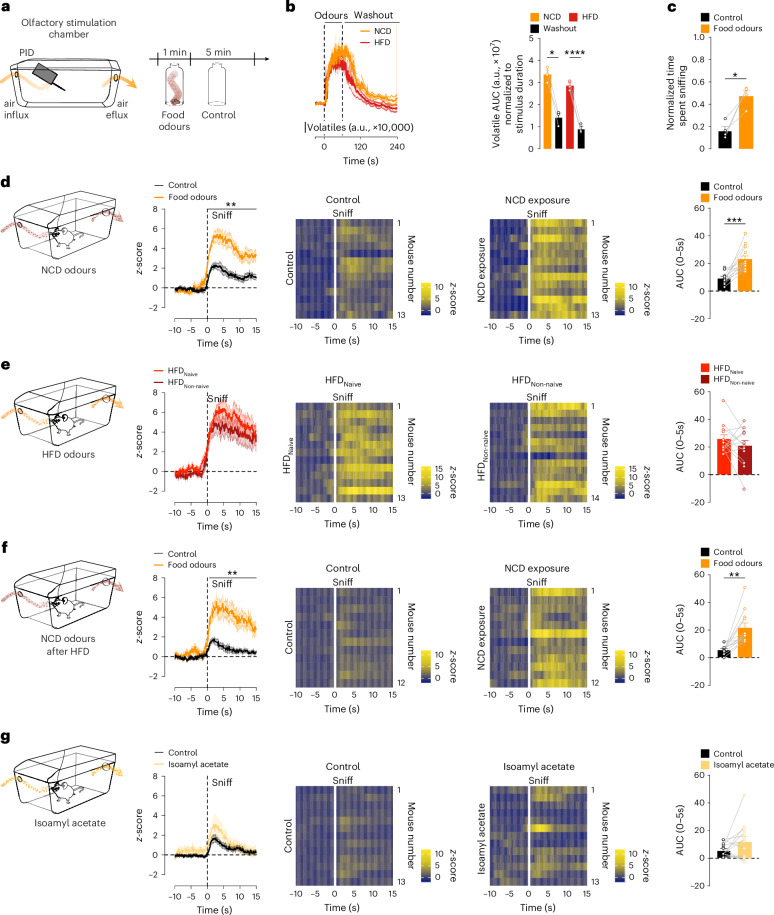
Olfactory perception of food increases the activity of MS^VGLUT2^ neurons independent of palatability. **a**, Schematic of the olfactory stimulation chamber allowing for the controlled influx and efflux of odorized air through an olfactometer-based system. A PID measured the amount of volatiles introduced into the system. **b**, Volatile concentration in the olfactory stimulation chamber during 1 min of food odour exposure of either NCD (orange) or HFD (red) followed by 5 min of exposure to the control odour (empty bottle; black; time curve: *n* = 3 trials; AUC: repeated-measures one-way ANOVA; *n* = 3 trials; *P* = 0.0002, *P* = 0.001). a.u., arbitrary units. **c**, Time spent sniffing in the olfactory stimulation chamber during food odour exposure (NCD; orange) or control exposure (black) normalized to the duration of stimulation (repeated-measures one-way ANOVA; *n* = 4 mice, three repetitions of food odours exposure; *P* = 0.0387). **d**–**g**, Calcium dynamics and AUC of MS^VGLUT2^ neurons aligned to the onset of the first sniff in response to NCD odours or control exposure (empty bottle; time curve: paired two-way ANOVA, Sidak post hoc; *n* = 13 mice; AUC: paired two-tailed Student’s *t*-test; *n* = 13 mice; *P* = 0.0006; **d**), HFD exposure when mice were naive (HFD_Naive_) or not naive to HFD (HFD_Non-naive_) (time curve: paired two-way ANOVA, Sidak post hoc; *n* = 13 mice for HFD_Naive_, *n* = 15 mice for HFD_Non-naive_; AUC: paired two-tailed Student’s *t*-test; *n* = 13 mice for HFD_Naive_, *n* = 15 mice for HFD_Non-naive;_ **e**), NCD or control re-exposure in HFD_Non-naive_ mice (time curve: paired two-way ANOVA, Sidak post hoc; *n* = 12 mice; AUC: paired two-tailed Student’s *t*-test; *n* = 12 mice; *P* = 0.0014; **f**) and isoamyl acetate or control exposure (time curve: paired two-way ANOVA, Sidak post hoc; *n* = 13 mice; AUC: paired two-tailed Student’s *t*-test; *n* = 13 mice; **g**). Lines on top of graphs indicate time points with significant differences in calcium signal between conditions. Data are represented as the mean ± s.e.m. *P ≤ 0.05, **P ≤ 0.01, ***P ≤ 0.001, ****P ≤ 0.0001. Panels **d**–**g** created using BioRender.com. Source data

Given that food sensory perception triggers MS^VGLUT2^ neuronal activity, we next examined the contribution of food odours to these responses. To selectively test the response to food odours without exposing mice to other food sensory cues or food-predicting cues, we developed an olfactory stimulation chamber (Fig. 4a). To avoid the animal associating the experimental set-up with food-related stimuli, we used the standard home cage as a basis for the olfactory stimulation chamber. An olfactometer-based odour delivery system was built to create an influx of odorized air into the cage, and a pump was used to generate an air efflux, ensuring a rapid air exchange. The exact concentration of volatiles present in the olfactory stimulation chamber was measured via a photoionization detector (PID; Fig. 4b). The volatile concentration of both NCD and high-fat diet (HFD) increased rapidly following the start of the odour influx and returned to basal 1 min after switching to control (Fig. 4b). The increased volatile concentration was accompanied by increased sniffing behaviour, that is, mice sniffing in the air, in mice exposed to food odours (NCD) compared to control (Fig. 4c). Hence, the olfactory stimulation chamber allows the exposure of mice to food odours in a timely and precise manner without interfering with other sensory modalities or generating food–predictive cue associations.

**Extended Data Fig. 4:**
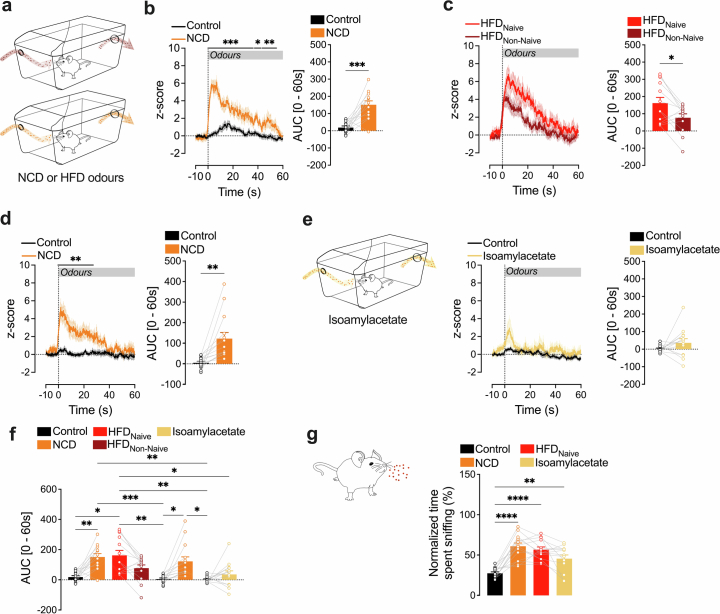
MS^VGLUT2^ neurons are rapidly activated upon food-odour exposure onset. **a)** Schematic of normal chow diet (NCD) and high-fat diet (HFD) odour exposure during *in vivo* fibre photometry calcium recording. MS^VGLUT2^ calcium dynamics and area under the curve (AUC) aligned to the onset of the stimulus in response to **b)** control odour (empty bottle) and NCD odour exposure [time curve: two-way ANOVA, Sidak post-hoc; n = 14 mice] [AUC: Paired two-tailed Student’s t-test; n = 14 mice; p = 0.0003], **c)** HFD odour exposure in mice naive (HFD_Naive_) or not to HFD (HFD_Non-Naive_) [time curve: two-way ANOVA, Sidak post-hoc; n = 14 mice] [AUC: Paired two-tailed Student’s t-test; n = 14 mice; p = 0.0119], **d)** control odour and NCD odour exposure in HFD_Non-Naive_ mice [time curve: two-way ANOVA, Sidak post-hoc; n = 14 mice] [AUC: Paired two-tailed Student’s t-test; n = 14 mice; p = 0.0023] and **e)** control odour and isoamylacetate exposure [time curve: two-way ANOVA, Sidak post-hoc; n = 13 mice] [AUC: Paired two-tailed Student’s t-test; n = 13 mice]. **f)** AUC quantification comparison of control (black), NCD (orange), HFD (red) and isoamylacetate (yellow) odour-induced MS^VGLUT2^ neuronal activation [mixed-effects multiple comparison analysis; n = 13 mice exposed to isoamylacetate odours, n = 14 mice exposed to control, NCD, HFD odours]. **g)** Time spent sniffing during control (black), NCD (orange), HFD (red) and isoamylacetate (yellow) exposure normalized to the duration of the stimulus [paired one-way multiple comparison ANOVA, Tukey post-hoc; n = 13 mice; p < 0.0001, p < 0.0001, p = 0.0087]. Data are represented as mean ± SEM. *p ≤ 0.05, **p ≤ 0.01, ***p ≤ 0.001 and, ****p ≤ 0.0001. Panels **a**, **e** and **g** created using BioRender.com. Source data

Utilizing the olfactory stimulation chamber, we exposed fasted mice to the odour of an NCD, which triggered a potent increase in MS^VGLUT2^ neuronal activity compared with exposure to the control (Fig. 4d; MS^VGLUT2^ neuronal activity aligned to the first sniff and Extended Data Fig. 4a,b; MS^VGLUT2^ neuronal activity aligned to the onset of odour influx). Analysis of MS^VGLUT2^ neuronal responses throughout the odour stimulation (Extended Data Fig. 4b) revealed that the mean MS^VGLUT2^ neuronal activity peaked at 4 s after the onset of odour exposure and gradually declined to basal levels by the end of the exposure (Extended Data Fig. 4b).

Because the sensory regulation of other neuronal populations, such as AgRP or POMC neurons, is directly contingent on the food caloric content and this process is based on learned responses^12,13,20,45^, we next assessed whether the response of MS^VGLUT2^ neurons to food odours was dependent on the caloric density. HFD odour exposure in mice naive to HFD, that is, HFD_Naive_, triggered a potent MS^VGLUT2^ neuronal activation (Fig. 4e and Extended Data Fig. 4c) that did not significantly differ from the response evoked by the NCD odour (Extended Data Fig. 4f). To test if previous HFD ingestion could alter this response, we repeated the experiments in mice not naive to HFD (HFD_Non-naive_), that is, lean mice that consumed small amounts of HFD a few days before the experiments (Fig. 4e). HFD odour exposure in HFD_Non-naive_ mice heightened MS^VGLUT2^ neuronal activation to the same magnitude as in HFD_Naive_ mice (Fig. 4e and Extended Data Fig. 4c,f). As exposure to HFD can decrease the neuronal response to a less palatable food such as NCD in the hypothalamus^13,23^, we repeated the characterization of NCD odour response in HFD_Non-naive_ mice (Fig. 4f and Extended Data Fig. 4d,f). The NCD odour-induced activation of MS^VGLUT2^ neurons was similar in HFD_Naive_ and HFD_Non-naive_ mice, revealing that prior food experience does not influence MS^VGLUT2^ neuronal response to food odours.

**Extended Data Fig. 5:**
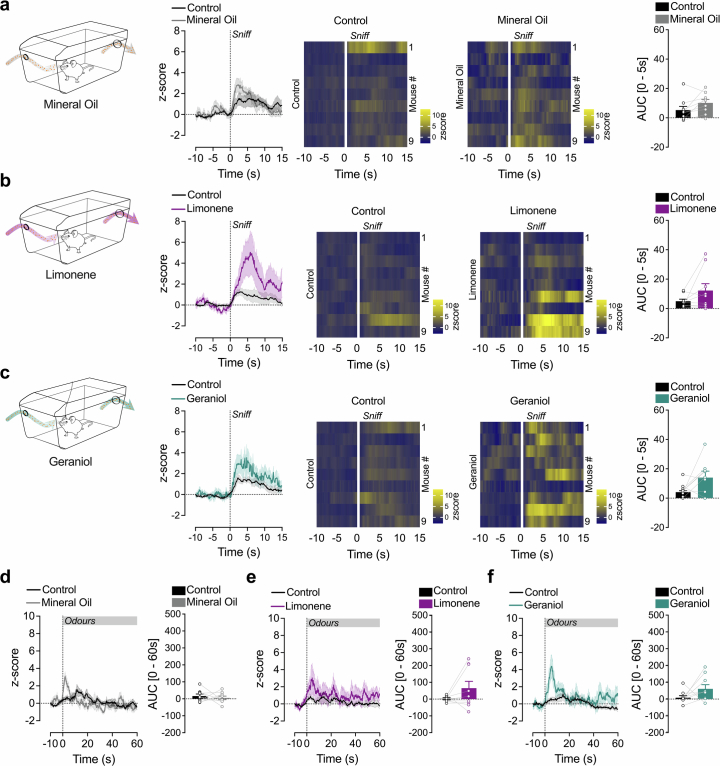
MS^VGLUT2^ neurons are not activated by non-food related odorants. **a-c)** Calcium dynamics and area under the curve (AUC) of MS^VGLUT2^ neurons aligned to the onset of the first sniff in response to **a)** mineral oil compared to control exposure (empty bottle) [AUC: paired two-tailed Student’s t-test; n = 9 mice], **b)** limonene compared to control exposure (empty bottle) [AUC: paired two-tailed Student’s t-test; n = 9 mice], or **c)** geraniol compared to control exposure (empty bottle) [AUC: paired two-tailed Student’s t-test; n = 9 mice]. **d-f)** Calcium dynamics and area under the curve (AUC) of MS^VGLUT2^ neurons aligned to the onset of the odour exposure to **d)** mineral oil compared to control exposure (empty bottle) [AUC: paired two-tailed Student’s t-test; n = 9 mice], **e)** limonene compared to control exposure (empty bottle) [AUC: paired two-tailed Student’s t-test; n = 9 mice], or **f)** geraniol compared to control exposure (empty bottle) [AUC: paired two-tailed Student’s t-test; n = 9 mice]. Data are represented as mean ± SEM. Panels **a**–**c** created using BioRender.com. Source data

Additionally, to investigate if the responses of MS^VGLUT2^ neurons to food odours can be generalized to other appetitive odours, we exposed mice to air odorized with isoamyl acetate, an odorant commonly used for its positive valence^46,47^ (Fig. 4g). Unlike NCD and HFD odours, isoamyl acetate did not elicit changes in MS^VGLUT2^ neuronal activity compared to exposure to the control (Fig. 4g and Extended Data Fig. 4e,f). Exposure to isoamyl acetate increased sniffing behaviour to the same extent as food odours, revealing that the animal did perceive the odour (Extended Data Fig. 4g). To further expand on the olfactory selectivity of MS^VGLUT2^ neuronal activity, we exposed fasted mice to innately neutral (mineral oil; Extended Data Fig. 5a,d), appetitive (limonene; Extended Data Fig. 5b,e) or aversive (geraniol; Extended Data Fig. 5c–f) odorants. None of these odorants significantly modulated MS^VGLUT2^ neuronal activity (Extended Data Fig. 5a–f), further pointing towards food odour-specific responses.

**Fig. 5:**
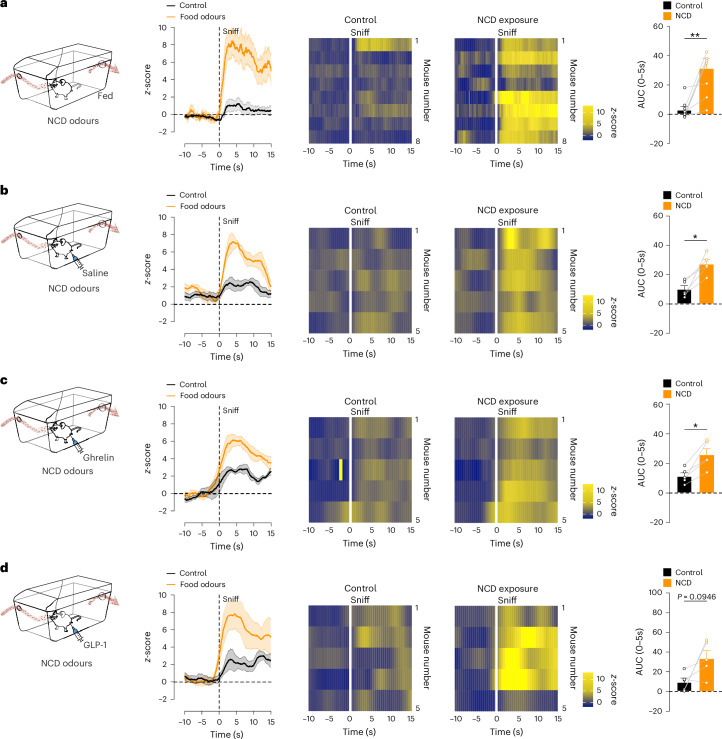
The nutritional status does not affect food odour-induced activity of MS^VGLUT2^ neurons. **a**–**d**, Calcium dynamics and AUC of MS^VGLUT2^ neurons aligned to the onset of the first sniff in response to NCD odours or control exposure (empty bottle), in random fed mice (time curve: paired two-way ANOVA, Sidak post hoc; *n* = 8 mice; AUC: paired two-tailed Student’s *t*-test; *n* = 8 mice; *P* = 0.054; **a**), after intraperitoneal (i.p.) saline injection (time curve: paired two-way ANOVA, Sidak post hoc; *n* = 5 mice; AUC: paired two-tailed Student’s *t*-test; *n* = 5 mice; *P* = 0.0130; **b**), after i.p. ghrelin injection (time curve: paired two-way ANOVA, Sidak post hoc; *n* = 5 mice; AUC: paired two-tailed Student’s *t*-test; *n* = 5 mice; *P* = 0.0127; **c**) and after i.p. GLP-1 injection (time curve: paired two-way ANOVA, Sidak post hoc; *n* = 5 mice; AUC: paired two-tailed Student’s *t*-test; *n* = 5 mice; **d**). Data are represented as the mean ± s.e.m. *P ≤ 0.05, **P ≤ 0.01, ***P ≤ 0.001, ****P ≤ 0.0001. Panels **a**–**d** created using BioRender.com. Source data

**Extended Data Fig. 6:**
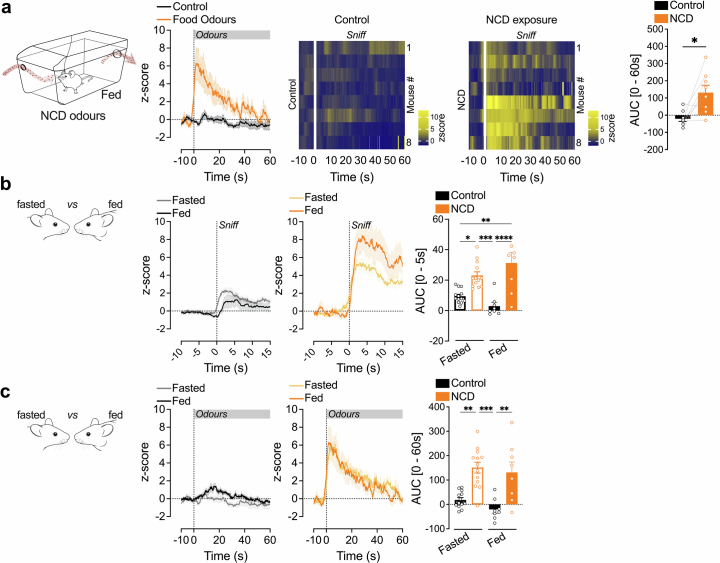
MS^VGLUT2^ neuronal activity is not affected by metabolic status. **a)** MS^VGLUT2^ calcium dynamics and area under the curve (AUC) aligned to the onset of the stimulus in response to control odour (empty bottle) and NCD odour exposure in random fed mice [AUC: paired two-tailed Student’s t-test; n = 8 mice]. **b-c)** Comparison of MS^VGLUT2^ calcium dynamics and area under the curve (AUC) aligned to **b)** the onset of a sniff and **c)** the onset of stimulus in response to control odour (empty bottle) and NCD odour exposure in mice fasted overnight for 16 hours or in random fed mice [AUC: two-way multiple comparisons ANOVA, Tukey post-hoc; n = 8 fed mice, n = 13 fasted mice]. Data are represented as mean ± SEM. *p ≤ 0.05, **p ≤ 0.01, ***p ≤ 0.001 and, ****p ≤ 0.0001. Mouse images in **b** and **c** adapted from SciDraw under a Creative Commons license CC BY 4.0. Panel **a** created using BioRender.com. Source data

**Extended Data Fig. 7:**
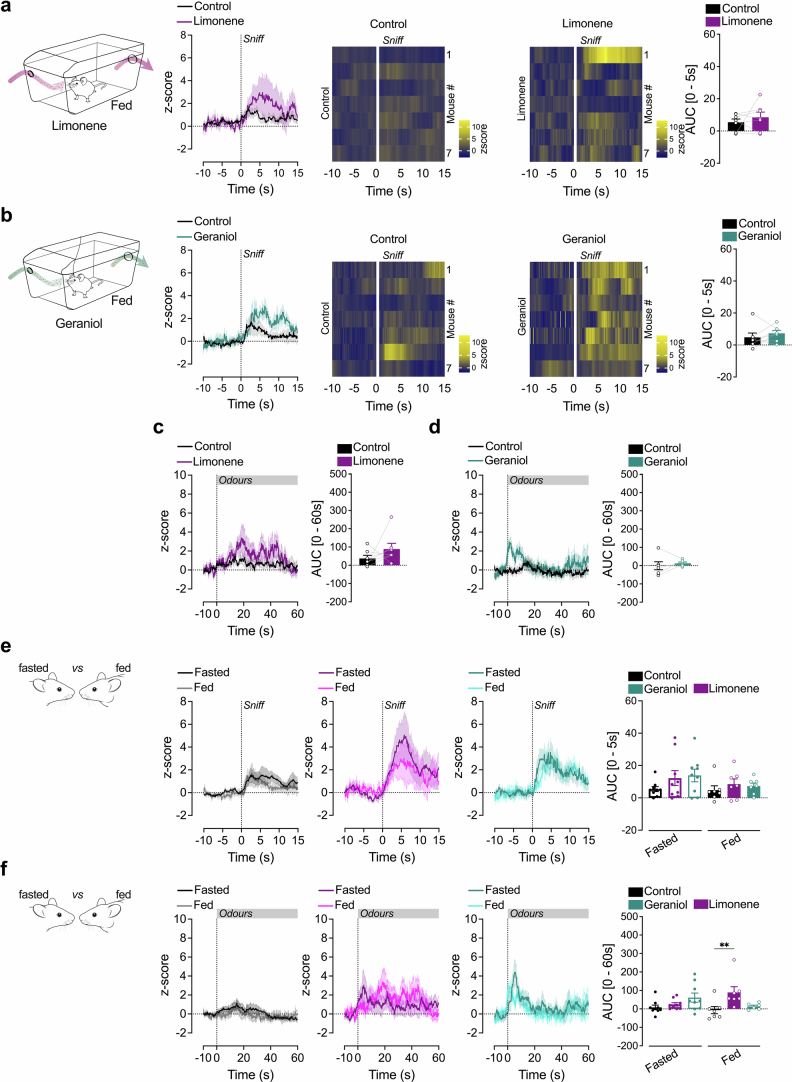
MS^VGLUT2^ neuronal activity in response to non-food related odours is not altered by metabolic status. **a-b)** Calcium dynamics and area under the curve (AUC) of MS^VGLUT2^ neurons aligned to the onset of the first sniff of random fed mice in response to **a)** limonene compared to control exposure (empty bottle) [AUC: paired two-tailed Student’s t-test; n = 7 mice], or **b)** geraniol compared to control exposure (empty bottle) [time curve: paired two-way ANOVA, Sidak post-hoc; n = 7 mice] [AUC: paired two-tailed Student’s t-test; n = 7 mice]. **c-d)** Calcium dynamics and area under the curve (AUC) of MS^VGLUT2^ neurons aligned to the onset of the odour exposure of random fed mice to **c)** limonene compared to control exposure (empty bottle) [time curve: paired two-way ANOVA, Sidak post-hoc; n = 7 mice] [AUC: paired two-tailed Student’s t-test; n = 7], or **d)** geraniol compared to control exposure (empty bottle) [time curve: paired two-way ANOVA, Sidak post-hoc; n = 7 mice] [AUC: paired two-tailed Student’s t-test; n = 7 mice]. **e-f)** Comparison of MS^VGLUT2^ calcium dynamics and area under the curve (AUC) aligned to **e)** the onset of a sniff and **f)** the onset of stimulus in response to control odour (empty bottle, black), limonene (purple) and geraniol (turquoise) exposure in mice fasted overnight for 16 hours or in random fed mice [time curve: two-way ANOVA, Sidak post-hoc; n = 7 fed mice, n = 9 fasted mice] [AUC: paired two-tailed Student’s t-test; n = 7 fed mice, n = 9 fasted mice]. Lines on top of graphs indicate time points with significant differences in calcium signal between conditions. Data are represented as mean ± SEM. *p ≤ 0.05, **p ≤ 0.01, ***p ≤ 0.001 and, ****p ≤ 0.0001. Mouse images in **e** and **f** adapted from SciDraw under a Creative Commons license CC BY 4.0. Panels **a** and **b** created using BioRender.com. Source data

To test whether the food odour-induced activation of MS^VGLUT2^ neurons is dependent on the nutritional state, we repeated the food odour exposure in fed animals. As observed in fasted mice (Fig. 4d), MS^VGLUT2^ neuronal activity rapidly increased following exposure to NCD odours in random fed mice (Fig. 5a and Extended Data Fig. 6a). The magnitude of MS^VGLUT2^ neuronal activation did not significantly differ between fasted and fed conditions (Extended Data Fig. 6b,c). To further rule out any influence of the nutritional state on the MS^VGLUT2^ neuronal activity dynamics, we recorded the food odour responses of MS^VGLUT2^ neurons in mice intraperitoneally injected with hunger and satiety hormones (Fig. 5b–d). Exposure to food odours activated MS^VGLUT2^ neurons to the same extent in mice injected with either saline or the hunger-promoting hormone ghrelin (Fig. 5c). MS^VGLUT2^ neuronal activity following NCD odour exposure was analogous, although not significant, in mice injected with saline or the satiety-related hormone glucagon-like peptide-1 (GLP-1; Fig. 5d). Similarly, non-edible odorants such as limonene and geraniol did not affect MS^VGLUT2^ neuronal activity in fed animals, as previously observed in fasted mice (Extended Data Fig. 7a–f). Altogether, these results suggest that the odour-evoked response of MS^VGLUT2^ neurons, while specific to food, is not contingent on the metabolic status of an animal.

### The OB sends direct input to MS^VGLUT2^ neurons

**Fig. 6:**
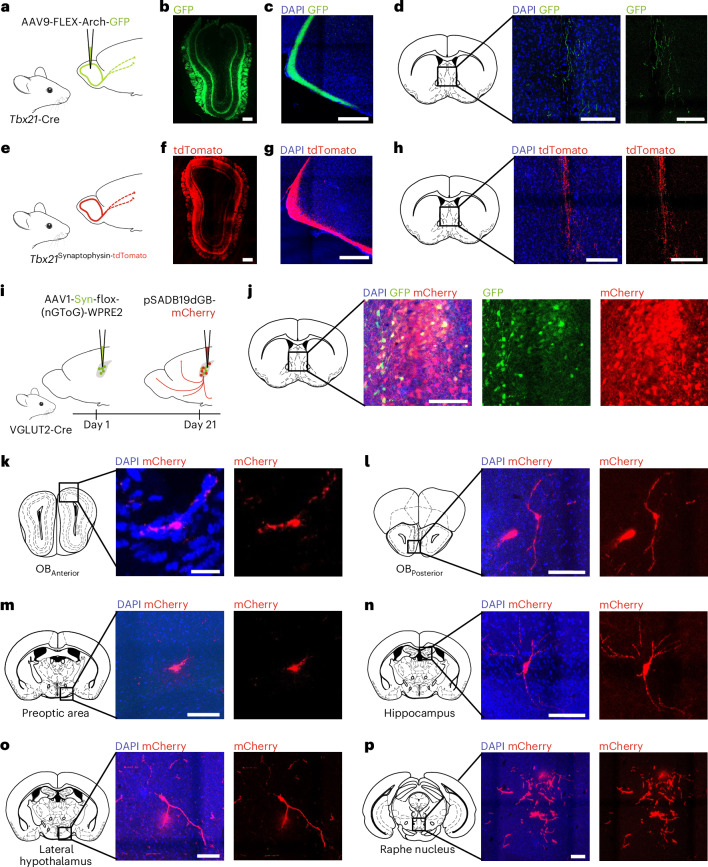
The MS receives direct projections from the OB. **a**, Schematic of anterograde AAV-based mapping of MTC projections in *Tbx21*-Cre mice. **b**–**d**, Representative images of AAV9-FLEX-Arch-GFP-expressing cells (GFP, green; DAPI, blue) in the OB (scale bar, 250 µm; **b**), the LOT (scale bar, 100 µm; **c**) and the MS (scale bars, 100 µm; **d**). **e**, Schematic of anterograde mapping of MTC projections in *Tbx21*^synaptophysin-tdTomato^ mice. **f**–**h**, Representative images of synaptophysin-tdTomato-expressing cells (tdTomato, red; DAPI, blue) in the OB (scale bar, 250 µm; **f**), the LOT (scale bar, 100 µm; **g**) and the MS (scale bars, 100 µm; **h**). **i**, Schematic of retrograde rabies-based monosynaptic tracing of projections onto glutamatergic neurons in the MS in VGLUT2-Cre mice. **j**, Representative images of the injection site, that is, the MS (GFP, green; mCherry, red; DAPI, blue; scale bar, 100 µm). **k**–**p**, Representative images of cells projecting onto MS^VGLUT2^ neurons (mCherry, red; DAPI, blue) in the anterior (scale bar, 50 µm; **k**) and posterior (scale bar, 100 µm; **l**) OB, the preoptic area (scale bar, 100 µm; **m**), the hippocampus (scale bar, 100 µm; **n**), the lateral hypothalamus (scale bar, 100 µm; **o**) and the raphe nucleus (scale bar, 100 µm; **p**). Mouse images in **a**, **e** and **i** adapted from SciDraw under a Creative Commons license CC BY 4.0.

Having uncovered that MS^VGLUT2^ neurons are sensitive to food odours, we next determined whether these effects could be mediated through direct inputs from the OB-derived axonal projections by characterizing the projection pattern of MTCs. We first used a Cre-dependent AAV-based approach in *Tbx21*-Cre mice to express a GFP reporter coupled to Arch^48^, a protein commonly used for investigating long-range neuronal projections (Fig. 6a). GFP immunoreactivity was observed in the soma of MTCs in the OB (Fig. 6b) as well as in the lateral olfactory tract (LOT) and classical projection sites such as the amygdala (Fig. 6c). In addition, MTC projections were also present in the septal region, where their innervation pattern was restricted to the MS (Fig. 6d). To corroborate the existence of direct projections from the OB to the MS, we mapped MTC synaptic termini in *Tbx21*^Synaptophysin-tdTomato^ mice, in which synaptophysin, a protein belonging to the presynaptic vesicle exocytosis machinery, was flanked by loxP sites and fused to a fluorescent tdTomato marker (Fig. 6e,f). Among various MTC projection sites such as the LOT and the amygdala (Fig. 6g), *Tbx21*^Synaptophysin-tdTomato^ mice also revealed direct projections from the OB to the MS (Fig. 6h).

To further examine whether MS^VGLUT2^ neurons receive direct projections from the OB, we performed mapping of monosynaptic afferents to MS^VGLUT2^ neurons using an EnvA pseudotyped, G-deleted rabies tracing approach (Fig. 6i,j). This rabies virus-based synaptic connectivity tracing revealed the presence of pSADB19dGB-mCherry-positive cells in the MTC layer of the anterior dorsal OB (Fig. 6k) and posterior medial OB (Fig. 6l); further supporting the existence of direct monosynaptic afferents from the OB to MS^VGLUT2^ neurons. In addition, pSADB19dGB-mCherry-positive cells were also found in regions previously identified^43^ to send direct projections to MS^VGLUT2^ neurons such as the preoptic area (Fig. 6m), the hippocampus (Fig. 6n), the lateral hypothalamus (Fig. 6o) and the raphe nucleus (Fig. 6p). Collectively, these analyses discovered direct connections between the OB and MS^VGLUT2^ neurons.

### Stimulation of OB→MS projections decreases food consumption

**Fig. 7:**
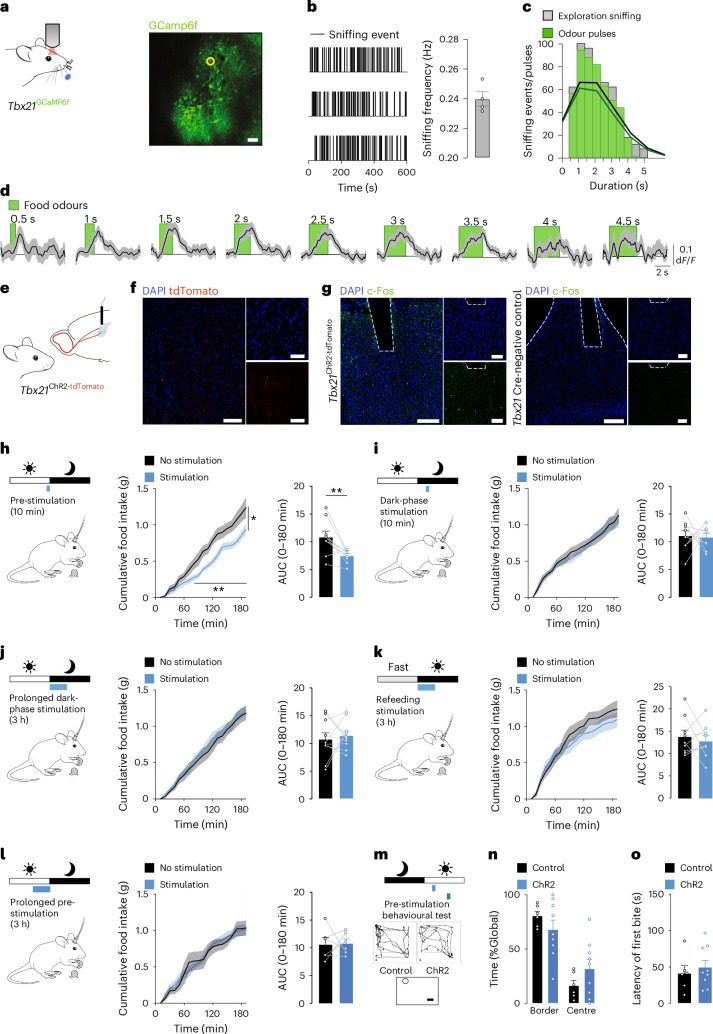
Acute activation of OB→MS projections before food ingestion reduces food intake. **a**, Schematic of 2P calcium imaging in the dorsal OB of awake head-fixed *Tbx21*^GCaMP6f^ animals and representative image of GCaMP6f fluorescence. Scale bar, 50 µm. **b**, Sniffing behaviour of fasted C57BL/6N mice exposed to food odours and average sniffing frequency (*n* = 4 mice). **c**, Distribution of sniffing exploration events (0.5–4.5 s) monitored in the home cage (grey, *n* = 4 mice) and odour pulse duration in imaging experiments (green). **d**, Example calcium activity traces of a representative neuron (region of interest indicated by the yellow circle in **a**) in response to food odours (green) of varying duration depicted as the mean ± s.e.m. (*n* = 10–130 trials). **e**, Schematic of optogenetic stimulation of OB→MS projections in *Tbx21*^ChR2-tdTomato^ mice. **f**, Representative images of MTC projections in the MS of *Tbx21*^ChR2-tdTomato^ mice (tdTomato, red; DAPI, blue). Scale bars, 100 µm. **g**, Representative images of photostimulation-induced FOS immunoreactivity in *Tbx21*^ChR2-tdTomato^ and *Tbx21*-Cre-negative control mice (FOS, green; DAPI, blue). Scale bars, 100 µm. **h**–**l**, Cumulative food intake and AUC of optogenetically stimulated *Tbx21*^ChR2-tdTomato^ mice (stimulation, blue line) and control mice (no stimulation, grey/black line), following 10 min pre-stimulation before the dark cycle (time curve: paired two-way ANOVA, Sidak post hoc; *n* = 8 mice; AUC: paired two-tailed Student’s *t*-test; *n* = 8 mice; *P* = 0.0078; **h**), following a 10-min stimulation during the dark cycle (time curve: paired two-way ANOVA; Sidak post hoc; *n* = 8 mice; AUC: paired two-tailed Student’s *t*-test; *n* = 8 mice; **i**), during a 180-min stimulation during the dark cycle (time curve: two-way ANOVA, Sidak post hoc; *n* = 10 mice; AUC: paired two-tailed Student’s *t*-test; *n* = 10 mice; **j**), during a 180-min stimulation after a 16-h overnight fast during the light cycle (time curve: paired two-way ANOVA, Sidak post hoc; *n* = 9 mice; AUC: paired two-tailed Student’s *t*-test; *n* = 9 mice; **k**) and following 180 min pre-stimulation before the dark cycle (time curve: paired two-way ANOVA, Sidak post hoc; *n* = 7 mice; AUC: paired two-tailed Student’s *t*-test; *n* = 7 mice; **l**). **m**, Behavioural test 60 min following a 10-min stimulation of *Tbx21*^ChR2-tdTomato^ (ChR2) and *Tbx21*-Cre-negative (control) animals. **n**, Open field test depicted as average time spent per area (border versus centre; two-way ANOVA, Tukey post hoc; *n* = 6 control mice, *n* = 9 ChR2 mice). **o**, Food finding test (unpaired two-tailed Student’s *t*-test; *n* = 6 control mice, *n* = 9 ChR2 mice). Data are represented as the mean ± s.e.m. *P ≤ 0.05, **P ≤ 0.01. Mouse images in **a**, **e** and **h**–**l** adapted from SciDraw under a Creative Commons license CC BY 4.0. Source data

**Extended Data Fig. 8:**
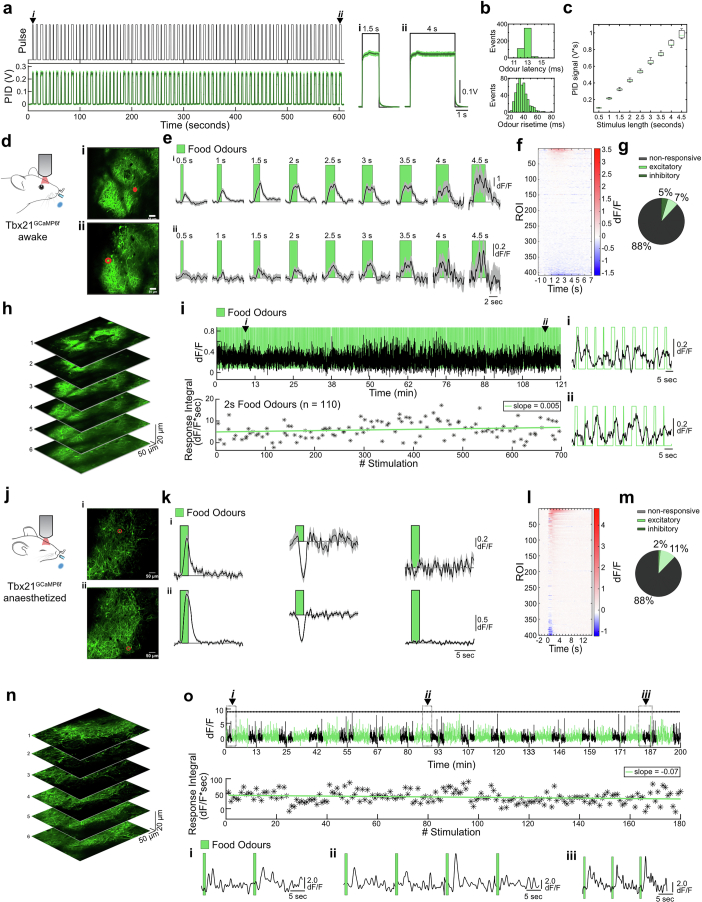
MTCs sustain their food odour-induced neuronal activation up to 3 hours. **a)** Left: Food odour stimulation paradigm. Top: odour valve commands, Bottom: food odour concentration. Right: Higher magnification of first (i) and last (ii) odour pulse average ± SEM [n = 3 trials]. **b)** Top: Distribution of latency times between valve command and odour detected by photoionisation detector (PID) [n = 480 presentations]. Bottom: Distribution of PID signal rise time [n = 480 presentations]. **c)** PID signal amplitude as a function of odour stimulus duration [n = 10-130 presentations]. **d)** GCaMP6f recording in awake mice and representative images of plane 1 (i) and 5 (ii) (an example neuron indicated in red). Scale bars: 50 µm. **e)** Average calcium activity traces of region of interest (ROIs) in response to food odour stimuli of varying duration shown ± SEM [10-130 trials per stimulus]. Neurons 1 and 2 correspond to ROIs indicated in (d). **f)** Average responses of all identified neurons [n = 408 ROIs from 2 animals, n = 110 stimulations, 1 s duration]. Timepoint 0 indicates the onset of odour presentation. **g)** Activity profiles of all neurons in response to food odours, separated into excitatory and inhibitory responses. **h)** GCaMP6f fluorescence from 6 stacked planes gained from volumetric imaging (total volume scanned=180 µm, z-distance=30 µm, maximum projections of 3200 frames). **i)** Top: Calcium activity time series of an example ROI over the duration of the whole experiment with valve commands shown in green. Right: Segments of the time series at extended timescales at early (i) and late (ii) time points in the experiment. Bottom: Integrals of calcium activity in response to each 2 s-long trial of food odour stimulation [n = 110] fitted with linear regression shown in red (slope=0.005). **j)** GCaMP6f recording in anaesthetized mice and representative images of planes 3 (i) and 4 (ii) (example neuron indicated in red). Scale bars: 50 µm. **k)** Example activity of neurons in response to food odours. Depicted is the mean of 30-180 trials ± SEM. Neurons 1 and 2 correspond to ROIs indicated in (**j**). **l)** Averaged responses of all identified neurons [n = 401 ROIs from 2 animals, n = 180 stimulations]. Timepoint 0 indicates the onset of odour presentation. **m)** Activity profiles of all neurons in response to food odours, separated into excitatory and inhibitory responses. **n)** GCaMP6f fluorescence from 6 stacked planes gained from volumetric imaging in an anaesthetized animal (total volume scanned=150 µm, z-distance=25 µm, maximum projections of 3200 frames). **o)** Top: Calcium activity time series of an example neuron over the entire duration of the experiment with individual odour stimulations indicated by black dots. Food odour exposure segments of the trace are depicted in green. Below: Integrals of calcium activity in response to food odours fitted with linear regression [n = 180 stimulations]. Below: Segments of the time series at extended timescales at early (i), middle (ii) and late (iii) time points. Mouse images in **d** and **j** adapted from SciDraw under a Creative Commons license CC BY 4.0. Source data

Having identified that the MS receives direct projections from the OB, we next used optogenetics to functionally assess the role of OB→MS projections in feeding behaviour. To define an optogenetic stimulation paradigm mimicking natural MTC neuronal activity following detection of food odours before ingestion, we first characterized the firing dynamics of MTCs in response to food odours in vivo (Fig. 7a). Head-fixed two-photon (2P) calcium imaging was performed in mice expressing the genetically encoded calcium indicator GCaMP6f in MTCs, that is, *Tbx21*^GCaMP6f^ mice (Fig. 7a). To deliver odour pulses that mimic the natural sniffing pattern of mice exposed to food odours, the exact sniffing pattern of fasted C57BL/6N mice investigating food odours was characterized (Fig. 7b) and used as a basis to generate pulses of food odour-odorized air during 2P imaging (Fig. 7c,d and Extended Data Fig. 8a–c). Beyond providing a basis to generate an optogenetic stimulation paradigm mimicking the natural firing of MTCs in response to food odour exposure (Fig. 7d and Extended Data Fig. 8d,e,j,k), these results also revealed that only a small population of MTCs is activated by food odours (Extended Data Fig. 8f,g,l,m) and sustain their activity for a prolonged period of stimulation (Extended Data Fig. 8h,i,n,o).

To optogenetically activate OB→MS projections (Fig. 7e), we generated mice expressing the light-sensitive channelrhodopsin-2 (ChR2) in MTCs, that is, *Tbx21*^ChR2-tdTomato^ mice in which tdTomato-positive MTC fibres are present in the MS (Fig. 7f). Of note, light stimulation in *Tbx21*^ChR2-tdTomato^ mice led to FOS immunoreactivity in the MS, whereas light stimulation in Cre-negative control mice induced no or little FOS immunoreactivity (Fig. 7g), confirming increased downstream excitation following optogenetic stimulation of OB→MS projections.

**Extended Data Fig. 9:**
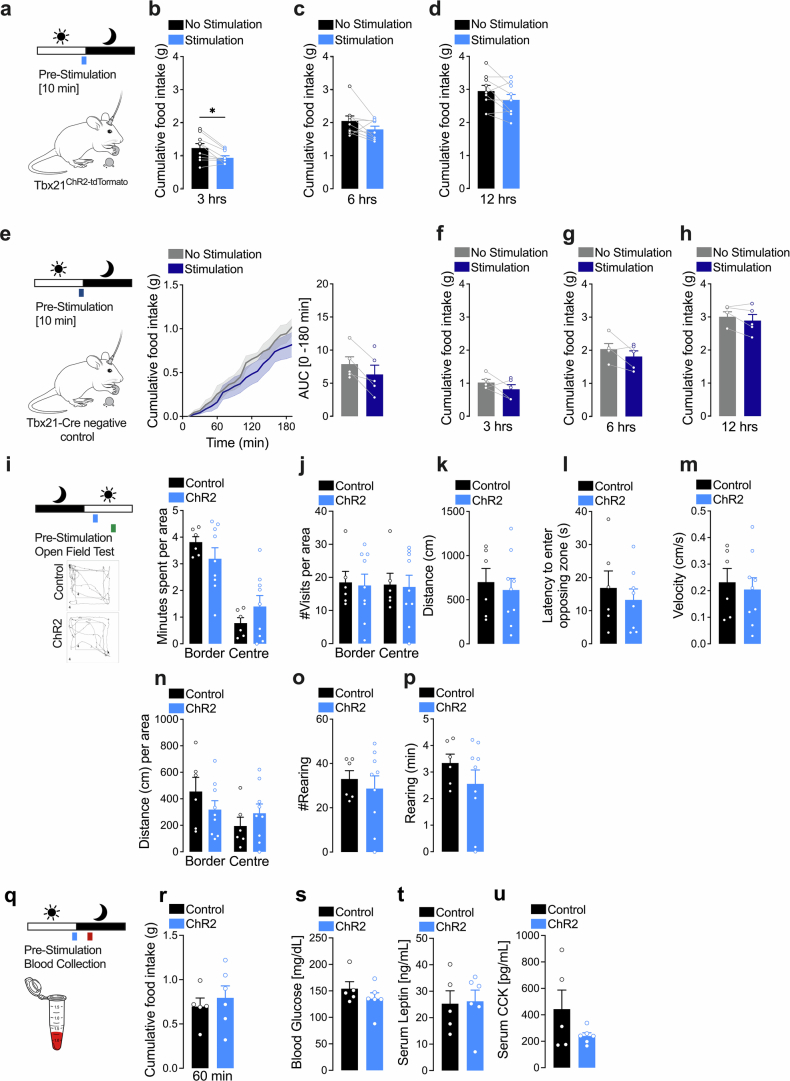
Optogenetic stimulation of OB→MS projections did not alter locomotion or anxiety-like behaviour. **a)** Schematic of the pre-stimulation protocol employed for OB→MS projections optogenetic stimulation in Tbx21^ChR-tdTomato^ (ChR2). **b-d)** Cumulative food intake of Tbx21^ChR2-tdTomato^ mice starting at the onset of dark cycle following a 10-minute pre-stimulation before dark cycle after **b)** three hours, **c)** six hours **d**) twelve hours [paired two-tailed Student’s t-test; n = 10 mice]. **e)** Cumulative food intake and AUC of Tbx21-Cre negative control animals starting at dark cycle onset following a 10-minute pre-stimulation before the dark cycle [time curve: two-way ANOVA, Sidak post-hoc; n = 5 mice] [AUC: two-tailed Student’s t-test; n = 5 mice]. **f-h)** Cumulative food intake of Tbx21-Cre negative control animals **f)** three hours, **g)** after six hours and **h)** after twelve hours after dark cycle onset following a 10-minute stimulation before dark cycle [two-tailed Student’s t-test; n = 5 mice]. **i-p)** Open field test 60 minutes after a 10-minute stimulation in Tbx21^ChR-tdTomato^ (ChR2) and Tbx21-Cre negative (control) mice depicted as **i)** minutes spent per area [two-way ANOVA, Tukey post-hoc; n = 6 Control mice, n = 9 ChR2 mice], **j)** number of visits per area [two-way ANOVA, Tukey post-hoc; n = 6 Control mice, n = 9 ChR2 mice], **k)** total distance travelled [two-tailed Student’s t-test; n = 6 Control mice, n = 9 ChR2 mice], **l)** latency to enter the opposing zone [two-tailed Student’s t-test; n = 6 Control mice, n = 9 ChR2 mice], **m)** velocity of travel [two-tailed Student’s t-test; n = 6-9], **n)** distance travelled per area [two-way ANOVA, Tukey post-hoc; n = 6 Control mice, n = 9 ChR2 mice], **o)** number and **p)** minutes of rearing [two-tailed Student’s t-test; n = 6 Control mice, n = 9 ChR2 mice]. **q-u)** Blood collection 60 minutes after a 10-minute stimulation in Tbx21^ChR-tdTomato^ (ChR2) and Tbx21-Cre negative (control) mice. **r**) Food intake, **s)** blood glucose levels, **t**) serum leptin and **u)** serum cholecystokinin (CCK) levels 60 minutes after a 10-minute stimulation [two-tailed Student’s t-test; n = 5 Control mice, n = 6 ChR2 mice]. Data are represented as mean ± SEM. Eppendorf image in **q** and mouse images in **a** and **e** adapted from SciDraw under a Creative Commons license CC BY 4.0. Source data

To mimic the naturally increased activity of the MS^VGLUT2^ population before food ingestion (Fig. 3f), we photostimulated OB→MS projections in random fed mice during the 10 min preceding the dark phase, that is, when mice naturally eat (Fig. 7h). Acute optogenetic stimulation of OB→MS projections significantly decreased cumulative food intake by 24% (Fig. 7h) compared to non-light-stimulated control mice. Food intake diminished after 70 min following the 10-min OB→MS stimulation and remained significantly lower up to 180 min (Extended Data Fig. 9a–d). In contrast, light stimulation had no effect on food intake in Cre-negative control animals (Extended Data Fig. 9e–h). Importantly, 10 min of acute stimulation of OB→MS projections during the dark phase did not alter food intake (Fig. 7i), revealing that the decreased feeding following acute stimulation of OB→MS projections is selectively driven by activation of this circuit before food ingestion (Fig. 7h).

Since the characterization of MTC responses to food odours revealed that this neuronal population is activated by food odours for up to 3 h (Extended Data Fig. 8n,o), we optogenetically stimulated OB→MS projections for 3 h during the dark phase in random fed mice (Fig. 7j) or following a fasting–refeeding protocol (Fig. 7k). Neither prolonged dark-phase stimulation nor prolonged refeeding stimulation of OB→MS projections altered food consumption (Fig. 7j,k). Prolonged 3-h optogenetic activation of OB→MS projections before the onset of the dark cycle did not influence food intake (Fig. 7l), further highlighting the selective effects of acute pre-stimulation in the initiation of anticipatory satiety processes. Furthermore, acute optogenetic stimulation of OB→MS projections did not influence the animal’s performance in either an open field test (Fig. 7m and Extended Data Fig. 9i–p) or a food finding test (Fig. 7n). In addition, acute optogenetic stimulation of OB→MS projections before the onset of the dark cycle (Extended Data Fig. 9q,r) did not significantly alter blood glucose levels (Extended Data Fig. 9s) or circulating levels of the satiety hormones leptin (Extended Data Fig. 9t) and cholecystokinin (Extended Data Fig. 9u). Collectively, these datasets reveal that the decreased food intake does not originate from increased anxiety, altered locomotion or olfactory acuity.

### OB→MS projections did not decrease feeding in obese mice

**Fig. 8:**
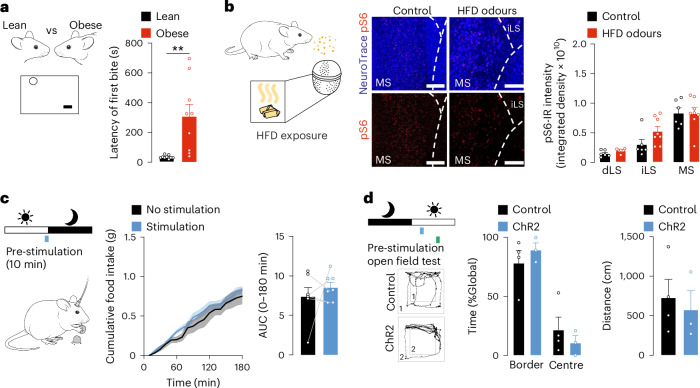
Diet-induced obesity blunts OB→MS anorexigenic effects. **a**, Food finding test in fasted control diet-fed (lean, black) and HFD-fed (obese, black) C57BL/6N mice (unpaired two-tailed Student’s *t*-test; *n* = 10 mice; *P* = 0.0037). **b**, Representative images of the MS and quantification comparison of food odour-induced (HFD) phosphorylated S6 (pS6)-IR in the dLS, iLS and MS in fasted C57BL/6N mice after 90 min (two-way ANOVA, Sidak post hoc; *n* = 6 mice for control, *n* = 8 mice for food odours; pS6, red; NeuroTrace, blue). Scale bars, 100 µm. **c**, Cumulative food intake and AUC following 10-min pre-stimulation before the dark cycle in optogenetically stimulated obese *Tbx21*^ChR2-tdTomato^ (stimulation, blue line) and control (no stimulation, grey/black line) mice (time curve: paired two-way ANOVA, Sidak post hoc; *n* = 7 mice; AUC: unpaired two-tailed Student’s *t*-test, *n* = 7 mice). **d**, Open field test 60 min following a 10-min stimulation of *Tbx21*^ChR2-tdTomato^ (ChR2) and *Tbx21*-Cre-negative (control) animals depicted as time spent per area (border versus centre; two-way ANOVA, Sidak post hoc; *n* = 3 control mice, *n* = 4 ChR2 mice) and total distance travelled (unpaired two-tailed Student’s *t*-test; *n* = 3 control mice, *n* = 4 ChR2 mice). Data are represented as mean ± s.e.m. **P ≤ 0.01. Mouse images in **a**–**c** adapted from SciDraw under a Creative Commons license CC BY 4.0. Source data

Having identified a feeding-regulatory neurocircuit, we next sought to investigate the functionality of this pathway in obesity. As previously reported^49,50^, olfactory acuity is impaired in diet-induced obese (DIO) mice, as revealed by decreased performance in a food finding test (Fig. 8a and Extended Data Fig. 10a). To assess the functionality of OB→MS projections in obesity, we reiterated experiments described above in DIO mice. Using phosphorylation of the ribosomal pS6 as a proxy for neuronal activity, a 90-min exposure to food odours did not trigger activation in the OB (Extended Data Fig. 10b) or in the MS (Fig. 8b) unlike occurring in lean NCD-fed mice (Fig. 2b and Extended Data Fig. 2b,c). Furthermore, while acute optogenetic stimulation of OB→MS projections before onset of the dark cycle dampened feeding in lean mice (Fig. 7h), a 10-min optogenetic activation of MTC projections within the MS before dark-phase onset did not affect feeding in DIO mice (Fig. 8c and Extended Data Fig. 10c–g). Acute optogenetic activation of OB→MS projections did not alter typical markers of anxiety as measured by an open field test (Fig. 8d and Extended Data Fig. 10l–r) and circulating levels of corticosterone (Extended Data Fig. 10s). Altogether, this dataset revealed that the MS is no longer responsive to food odours in DIO mice and that OB→MS projections failed to trigger anticipatory satiety in HFD-fed mice.

## Discussion

The discovery of the sensory regulation of feeding-regulatory neurons has been a major milestone in understanding the neurobiology of feeding. Although some aspects of the underlying regulatory principles have started to emerge, the contributions of distinct sensory modalities, their associated neural circuits and their roles in feeding regulation remain largely unclear. Here, we uncover a previously unreported sensory neurocircuit that co-ordinately integrates food odours to regulate impending food intake, highlighting the importance of anticipatory food sensory perception in the control of satiety.

Previous research has shown that food cues decrease the activity of the orexigenic AgRP neurons and increase the activity of the anorexigenic POMC and PVH^GLP-1R^ neurons^12,13,15,16^, suggesting that anticipation of caloric intake might prime satiety. Extending these studies, we present additional insights into the sensory regulation of hunger and satiety neurons by unravelling a neuronal population responding to food cues. Our investigation uncovers that food odours activate MS^VGLUT2^ neurons, a recently identified feeding-regulatory neuronal population^25–27^. MS^VGLUT2^ neurons exert anorexigenic effects as revealed by a reduction in food consumption following their chemogenetic or optogenetic activation^26,27^. While the sensory regulation of MS^VGLUT2^ neurons mirrors previous reports demonstrating that food cues activate other anorexigenic neuronal populations, the dynamics of MS^VGLUT2^ activity following food ingestion contrasts with those previously published. MS^VGLUT2^ neurons display a biphasic inverted modulation, where their neuronal activity is strongly and rapidly activated by food odours, followed by a decrease in activity directly following food ingestion. This contrasts with the activity pattern of other feeding neurons in response to food cues, which is gated towards their postprandial responses^12,13,15,16^. Therefore, our results suggest that while food odours activate MS^VGLUT2^ neurons, signals associated with the post-ingestive and satiety phases may inhibit them. This is consistent with recent findings by Xu et al. showing an excitatory histaminergic circuit from the hypothalamic tuberomammillary nucleus to MS^VGLUT2^ neurons, which rapidly inhibits neuronal activity at the onset of a feeding bout^27^. Indeed, while global inhibition of all MS^VGLUT2^ neurons does not significantly alter food consumption in mice^26^, optogenetic inhibition of the histaminergic MS^VGLUT2^-projecting neurocircuit increases food intake^27^.

In addition to demonstrating that MS^VGLUT2^ neurons integrate anticipatory olfactory cues, we also unveiled a previously uncharted connection between MS^VGLUT2^ and the glutamatergic MTCs of the OB. Through monosynaptic retrograde rabies mapping, we discovered direct projections from MTCs to MS^VGLUT2^ neurons, establishing a two-synapse neurocircuit from the external environment to these anorexigenic neurons. We found that MS^VGLUT2^ neurons are sensitive to various food-related olfactory cues but not to inedible objects or other odorants with a positive valence (for example, isoamyl acetate, limonene) or those with neutral or negative valence (for example, mineral oil, geraniol). This suggests that the OB→MS pathway is not broadly activated by all odours but exhibits a certain degree of selectivity for food odours. Accordingly, a recent study has shown that food-like odours, such as benzaldehyde, activate a different olfactory pathway than non-food odours, such as isoamyl acetate^51^, an observation mirroring the food odour-selective activation of MS^VGLUT2^ neurons. Furthermore, 2P calcium imaging of MTC neuronal activity revealed that only a subset of MTCs is activated by food odours, highlighting the existence of a distinct spatiotemporal pattern of activity in the OB during food odour perception^52–55^. Although this study did not examine the neurocircuitry downstream of MS^VGLUT2^ neurons, previous studies revealed that MS^VGLUT2^ projections to the PVH recapitulate the anorexigenic effects of MS^VGLUT2^ neurons^26^. Future studies will be required to identify the exact downstream targets of MS^VGLUT2^ neurons and to determine whether they contribute to the sensory regulation of AgRP and POMC neurons. In addition, since our study and previous studies selectively focus on male mice, it would be interesting to study these anorexigenic effects in female mice.

Our work established the functional relevance of this sensory neurocircuit by showing that optogenetic activation of olfactory inputs to the MS results in a significant decrease in food consumption. Importantly, the reduction in food intake was exclusively observed in response to acute optogenetic stimulation of this pathway before food consumption, highlighting the enduring role of sensory food perception on food intake^21,56–58^. These results are in line with the reported long-lasting reduction in food intake following a brief pre-stimulation of AgRP neurons^56^. Further supporting the role of olfactory food cues in satiety, Shen et al.^57^ demonstrated that exposure to limonene, a component of grapefruit oil, resulted in a significant reduction in food intake. These anticipatory anorexigenic effects support the hypothesis that the sensory modulation of feeding-regulatory neurons plays a role in anticipatory satiety, as proposed by others^21^. Thus, our study provides compelling evidence in favour of this hypothesis by demonstrating that acute stimulation of food-sensitive olfactory circuits selectively before food ingestion reduces feeding.

The existence of a mechanism limiting nutrient intake at the onset of a meal might provide strong benefits for an animal, both ethologically and physiologically. Anticipatory satiety might enable animals to strategically decrease predation risk by minimizing the temporal investment in feeding behaviour and hence exposure to predators and other threats. In line with this idea, AgRP neuronal activity is not only contingent on food-related cues but also modulated in response to predators, emphasizing an interplay between nutritional needs and predation risk^59,60^. Beyond predation risk reduction, anticipatory satiety may yield physiological advantages. Eating is considered a physiologically challenging event, leading to postprandial increases in blood glucose and lipid levels that must be tightly regulated by the body to avoid the onset of metabolic abnormalities such as diabetes and obesity^61^. Sensory food cues, therefore, allow animals to proactively adjust, mitigating the impact of eating on the body through compensatory responses, including hormonal release as well as priming of postprandial hepatic responses and lipolysis^8,10,58^. Our findings are aligned with recent publications demonstrating that the rate of food ingestion and onset of satiety is intricately regulated by the sensory perception of food^62,63^. Furthermore, Kim et al.^64^ recently reported that the anorexigenic effects of GLP-1 are partly mediated through pre-ingestive satiation both in mice and humans. This highlights the existence of parallel pathways by which organisms integrate food cues to prevent overconsumption. Thus, this study highlights the critical role of sensory perception in the regulation of food intake and provides mechanistic insights into the underlying circuits.

Consistent with our findings, the sensory regulation of feeding behaviour has also been reported in humans^2,65–68^. Notably, bypassing the cephalic phase by delivering food directly into the stomach fails to completely reduce appetite^65,67,68^. However, restoring food sensory cues by allowing individuals to chew but not swallow food during enteral feeding is sufficient to potentiate satiety^65^, highlighting the importance of food sensation in satiety in humans and thus emphasizing the translational relevance of our study.

While the primary focus of this study was investigating regulatory effects on feeding behaviour of MS^VGLUT2^ neurons, these also play pleiotropic roles in learning, memory and locomotion^69–72^. However, despite the documented activation of MS^VGLUT2^ neurons during locomotion^43,44,73,74^, the food odour-sensitive MS^VGLUT2^ neurons investigated here did not show changes in activity during locomotion, grooming or rearing behaviour, suggesting that distinct MS^VGLUT2^ subpopulations might respond to food odours and locomotion. Intriguingly, although the MS is known for its role in learning and memory^75–78^, MS^VGLUT2^ neuronal activity did not exhibit learned responses to food odours. In particular, responses to novel food odours were indistinguishable before and after acclimatization, in contrast to AgRP neuronal responses to food cues that are dictated by learned, experience-based mechanisms^12–14,20,45^. Similarly, unlike AgRP and POMC neurons, which show sensitivity to food cues contingent on caloric content and palatability^12,14,20,45^, MS^VGLUT2^ neurons showed consistent activation by food odours to the same order of magnitude irrespective of food palatability. In addition, the transient activation of MS^VGLUT2^ neurons in response to food odours was independent of the nutritional status. Thus, our findings suggest that this pathway may control food intake following sensory detection, regardless of the encountered food cue’s associated valence or hunger state of an animal.

Noteworthy, this study revealed that the OB→MS pathway may contribute to HFD overconsumption in obesity. The olfactory impairments observed in DIO mice coincide with a reduced neuronal activity in the OB^79–82^ and MS in response to a prolonged exposure to food odours, suggesting a compromised OB→MS pathway in obesity. Importantly, acute optogenetic activation of the OB→MS circuit in DIO mice before dark-phase onset fails to reduce food intake as observed in their lean counterparts. The proposed role of the OB→MS^VGLUT2^ circuit in regulating food intake in anticipation of nutrient ingestion raises the possibility that the acute anticipatory regulation of feeding behaviour is disrupted in the context of obesity. In support of this notion, mice with diphtheria toxin-induced ablation of olfactory sensory neurons exhibited increased food consumption when exposed to a fasting–refeeding paradigm, suggesting a deficiency in the initiation of olfactory anticipatory satiety mechanisms and responses^83^. Furthermore, although olfactory bulbectomy experiments remain controversial^84–87^, obesity-prone rats that underwent olfactory bulbectomy exhibited increased food intake^88^, supporting the notion of a diminished olfactory-based satiety in this model. Taken together, these findings suggest that anticipatory satiety is impaired in obesity, potentially contributing to the onset of diet-induced obesity.

In conclusion, this study reveals a sensory pathway that co-ordinately integrates food cues to control feeding behaviour by priming satiety. The discovery of this food-sensitive olfactory feeding circuit thus reveals a regulatory principle for the central control of energy homeostasis and may pave the way to a better understanding of the aetiology of metabolic disorders associated with olfactory dysfunction, such as obesity.

## Methods

### Animal care

Animal care, handling and experimental procedures were performed in accordance with protocols approved by the State Office for Nature, Environment and Consumer Protection of North Rhine-Westphalia, Germany. Permission to maintain and breed mice was issued by the Department of Environment and Consumer Protection – Veterinary Section, Cologne, North Rhine-Westphalia. Adult male mice were housed in individually ventilated cages at 22–24 °C with a 12-h light–dark cycle. Animals had ad libitum access to water and food at all times, unless stated otherwise. All experiments were performed on adult male mice aged 8–16 weeks, unless specified otherwise.

### Diets

All diets used were obtained from ssniff Spezialdiäten (ssniff, Germany). NCD (ssniff, V1554-703) was used as the control maintenance diet and contained 57 kJ% carbohydrate, 34 kJ% protein and 9 kJ% fat. For experiments involving HFD feeding, mice were fed a HFD (ssniff, E15742) containing 20 kJ% carbohydrates, 20 kJ% protein and 60 kJ% fat (lard) or corresponding control diet (ssniff, E15748) containing 67 kJ% carbohydrates, 20 kJ% protein and 13 kJ% fat.

### Mouse models

C57BL/6N mice were obtained from Charles River (Germany) and allowed to acclimatize to the animal facility for at least a week before the experiment. *Tbx21*-Cre (JAX stock, 024507)^40^, VGLUT2-IRES-Cre (JAX stock, 016963)^89^, Rosa26-CAG-LSL-Synaptophysin-tdTomato-WPRE (JAX stock, 012570)^90^ and Rosa26-CAG-LSL-ChR2(H134R)-tdTomato-WPRE mice (JAX stock, 012567)^91^ were originally obtained from The Jackson Laboratory and colonies were maintained at the Max Planck Institute for Metabolism Research, Cologne, Germany. The mice used in the experiments were obtained from in-house breeding. *Tbx21*^Synaptophysin-tdTomato^ (tg/wt; fl/fl or fl/wt) mice were obtained by breeding *Tbx21*-Cre tg/wt and R26-Synaptophysin-tdTomato fl/fl mice. *Tbx21*^ChR2-tdTomato^ (tg/wt; fl/fl) mice were obtained by breeding *Tbx21*-Cre tg/wt and R26-ChR2-tdTomato fl/fl mice. For 2P calcium imaging, *Tbx21*^GCaMP6f^ mice were obtained and maintained at the Francis Crick Institute by breeding *Tbx21*-Cre and Ai95(RCL-GCaMP6f)-D (GCaMP6f; JAX stock, 024105)^92^ mice.

### Stereotaxic surgeries

Two days before surgery, mice received tramadol (1 mg ml^−1^; Tramal) in drinking water. Mice were anaesthetized using 5% isoflurane and were placed in a stereotaxic apparatus (Kopf Instruments) under 2% isoflurane anaesthesia. An ointment (Bepanthen) was applied to the eyes to prevent desiccation. Before exposing the skull, the skin was sterilized with Octenisept (Schülke), and a local anaesthetic (Pierre Fabre) was applied to the incision site. A small hole was drilled over the target sites. Co-ordinates were chosen based on the Paxinos and Franklin brain atlas^93^. A 2-µl Hamilton syringe (Hamilton, 7002) and a syringe pump (Harvard Apparatus) were used to inject the virus at a rate of 40 nl min^−1^ and were subsequently left in place for 10 min before being slowly withdrawn. The skin was then sutured, and the mice were allowed to recover in their home cage. For postoperative care, mice were administered buprenorphine (0.1 mg per kg body weight i.p.) and meloxicam (5 mg per kg body weight, subcutaneous) and received tramadol in the drinking water (1 mg ml^−1^) for 3 days after surgery. Body weights were continuously monitored during recovery.

### Virus injections

#### MTC projection tracing

The OB of *Tbx21*-Cre (tg/wt) mice was targeted bilaterally (anteroposterior (AP): 4.5 mm, mediolateral (ML): 0.75 mm, dorsoventral (DV): −3.0 mm) with 500 nl AAV9-FLEX-ArCh-GFP (a gift from E. Boyden; Addgene plasmid 22222-AAV9; RRID: Addgene 22222)^48^. Two weeks after surgery, mice were deeply anaesthetized with ketamine–xylazine and transcardially perfused for further processing of the tissue.

#### MTC chemogenetic activation

The OB of *Tbx21*-Cre (tg/wt) mice was targeted bilaterally (AP: 4.5 mm, ML: 0.75 mm, DV: −3.0 mm) with 500 nl AAV9-hSyn-DIO-hM3D(Gq)-mCherry (a gift from B. Roth; Addgene plasmid 44361-AAV9; RRID: Addgene 44361)^94^. Two weeks after surgery, mice were deeply anaesthetized with ketamine–xylazine and transcardially perfused for further processing of the tissue.

#### Monosynaptic rabies tracing

The septal region of VGLUT2-Cre (tg/wt) mice was targeted along the midline (AP: 0.6 mm, ML: 0.0 mm, DV: −3.0 to 3.5 mm) with 150 nl AAV1-Syn-fl(nGToG)-WPRE (AAV-Ef1a-DIO-H2B-GFP-2A-oG-WPRE-hGH was a gift from E. Callaway; Addgene plasmid 74289 (ref. ^95^); RRID: Addgene 74289; and AAV-EF1a-DIO-HTB was a gift from E. Callaway; Addgene plasmid 44187; generated by the Charité Viral Core Facility) and 3 weeks later with 500 nl envA-1-SADB19dG-mCherry (viral particles were generated by the Charité Viral Core Facility^96,97^ and plasmids were gifted by E. Callaway; Addgene plasmids 32631, 32632, 32633 and 32634). Ten days after surgery, mice were deeply anaesthetized with ketamine–xylazine and transcardially perfused for further tissue processing.

### In vivo dual-colour fibre photometry calcium recording

#### Virus injection and fibre cannula placement

For in vivo fibre photometry recordings, 500 nl of AAV9.Syn.Flex.GCaMP6s.WPRE.SV40 (a gift from D. Kim and the GENIE Project, Addgene viral prep no. 100845-AAV9; RRID: Addgene 100845)^98^ was injected into the septal region (AP: 0.6 mm, ML: 0.0 mm, AP: −3 to −3.5 mm) of VGLUT2-Cre (tg/wt) mice. After virus delivery, an optical fibre (400-µm diameter, 0.48 NA, Doric Lenses) was implanted directly over the injection site and secured with dental acrylic (Super-Bond C&B, Sun Medical).

#### Training and acclimatization to the experimental set-up

Starting 2 weeks after surgery, mice were habituated to the patch cord for approximately 20 min per day in the appropriate behavioural set-up at least five times.

#### Data collection

Data were acquired using an RZ5P lock-digital processor controlled by Synapse software (Tucker-Davis Technologies) as previously described^99^.

#### Recording—food perception

Fibre photometry recording started 4 weeks after surgery to allow for optimal viral expression. Five weeks after surgery, mice were fasted overnight for 16 h after being placed into a novel clean cage without access to food and placed in the experimental set-up 2 h into the light cycle. After a 10-min acclimatization period during which the laser was on, mice were exposed to multiple non-edible objects (perforated metal teaball, wooden stick, a 15-ml Falcon tube lid) and the signal was recorded for 10 min. Afterwards, mice were exposed to NCD pellets and the signal was recorded for 10 min. As a final experiment, the signal was recorded for 10 min during food consumption.

#### Olfactory stimulation chamber

To selectively expose animals to odours, an olfactory stimulation chamber was constructed using a standard mouse cage (Tecniplast, GM500) to reduce the novelty of the behavioural set-up. Here, the chamber is connected to a filter (WH5 filter, Pentair OmniFilter) driven by a pump (APS300, Tetratec) via tubing in the upper-right corner of the chamber. In the opposite corner of the cage, tubing is connected to a brushless DC fan (40 × 40 × 10 mm, 24 V; HysiPrui), which is responsible for air removal. Polyethylene terephthalate bottles can be connected to the filter to allow the inflow of odorized air into the olfactory chamber. For olfactory exposure, four pellets of either NCD or HFD are dissolved in water for at least 10 min before the experiments in the respective polyethylene terephthalate bottles. Similarly, odorized air containing other odorants is introduced into the chamber by connecting a polyethylene terephthalate bottle containing two filter papers soaked in the solution (isoamyl acetate: 10^−^^8^ in mineral oil; limonene: 0.2% in mineral oil; geraniol: 21.26% in mineral oil; 100 μl on 1.5-cm^2^ filter paper). A constant inflow and outflow of air is ensured when a mouse is in the olfactory stimulation chamber. A PID (200 C: miniPID Fast Response Olfaction Sensor, Aurora Scientific) controlled by Synapse software (Tucker-Davis Technologies) was used to measure volatile concentration within the olfactory stimulation chamber during odorant stimulation. The collected data were subsequently analysed using MATLAB.

#### Recording—olfactory stimulation chamber

Five weeks after surgery, mice were either fasted overnight or random fed and placed in the olfactory stimulation chamber from 1 h to 5 h into the light cycle, where they were acclimatized to the connected pumps that inflated the chamber with ambient air for 10 min. After 5 min of exposure to an empty bottle (control), mice were exposed to the NCD odour for 1 min. This was followed by a 5-min exposure to the control to remove residual NCD odour. Mice were then exposed to the HFD odour to which they were naive, for 1 min, followed by a 5-min exposure to the control. Mice were exposed to small amounts of HFD in their home cage 6 weeks after surgery for 3 to 5 days to associate the smell of HFD with its caloric value. Mice were then fasted overnight and placed in the olfactory stimulation chamber during the light cycle. After a 10-min acclimatization period, the mice were exposed to the control for 5 min, followed by a 1-min exposure to HFD to which they were now non-naive. After a 5-min exposure to the control, mice were exposed to NCD for 1 min, followed by a final 5-min exposure to the control. One week later, overnight fasting was repeated, and recording was performed by exposing the mice to isoamyl acetate for 1 min. A separate cohort of NCD-fed mice were either fasted overnight or not fasted and exposed to non-food-related odours (mineral oil, limonene and geraniol) for 1 min as described above 5 weeks after surgery

#### Recording—hormone injection

Four weeks after surgery, mice were acclimatized to the experimental protocol. One week later, the mice were either fasted overnight or random fed and placed in the olfactory stimulation chamber from 1 to 5 h into the light cycle, where they were acclimatized to the connected pumps that inflated the chamber with ambient air for 15 min. After 5 min of exposure to an empty bottle (control), mice were subsequently injected with ghrelin (Tocris, 1465; 50 μg per kg body weight in 0.9% NaCl), GLP-1 (Bachem, H-6795.1000; 0.1 mg per kg body weight in 0.9% NaCl) or saline (0.9% NaCl, 1 ml per kg body weight) and recorded for 15 min. This was followed by a 1-min exposure to NCD odour and subsequently by a 3-min exposure to the control to remove residual NCD odour. Mice were then exposed to the HFD odour to which they were not naive for 1 min, followed by a 5-min exposure to the control.

#### Fibre photometry data analysis

All analyses of the fibre photometry data were performed using a custom MATLAB script. The initial 20 s of each recording were discarded to remove potential artefacts related to the activation of the light source. The data were then smoothed using a 20-Hz low-pass second-order (zero-lag) Butterworth filter before being down-sampled at 20 Hz to speed up processing. To correct for motion and bleaching artefacts, the isosbestic time series were linearly fitted to the signal and used as a reference to compute the Δ*F/F* = (signal − scaled isosbestic)/scaled isosbestic. Finally, the Δ*F/F* was *z*-scored to facilitate comparison across animals. Peri-event traces were extracted from a window spanning −300 s to +300 s around food ingestion manually identified from video recordings, −30 s to +120 s around food perception manually identified from video recordings as approaching and sniffing the food pellets and −30 s to +60 s around the first sniff during the different stimuli in the olfactory stimulation chamber manually identified from video recordings. To avoid spurious influences of the stimulus administered, the baseline was defined as the window from −60 s to −20 s for food ingestion, −30 s to 0 s for food perception and −30 s to 0 s for sniffing. Each peri-event trace was then *z*-scored using this baseline to calculate the mean and standard deviation. The mean (*z*-scored) trace was computed for each animal, and the AUC was calculated for specific time frames to compare between groups. For analysis after hormone injection, the initial 20 s of each recording were discarded and the data was smoothed using a low-pass 3 s moving mean before being down-sampled at 10 Hz to speed up processing. To correct for motion and bleaching artefacts, the isosbestic time series were linearly fitted to the signal and used as a reference to compute the Δ*F/F* = (signal − scaled isosbestic)/scaled isosbestic. Finally, the Δ*F/F* was *z*-scored to facilitate comparison across animals. Peri-event traces were extracted from a window spanning −30 s to +60 s around the first sniff during the different stimuli in the olfactory stimulation chamber manually identified from video recordings. To avoid spurious influences of the administered stimulus, the baseline was defined as the window from −30 s to 0 s for sniffing. Each peri-event trace was then *z*-scored using this baseline to calculate the mean and standard deviation. The mean (*z*-scored) trace was computed for each animal and the AUC was calculated for specific time frames to compare between groups. For display, a time frame of −10 s to 15 s was selected for first sniffs in response to odorized air (food odours, non-food-related odours) and control exposure (average response of all wash steps).

#### AAV expression and fibre placement validation

After experiments, mice were deeply anaesthetized with ketamine–xylazine and transcardially perfused for further processing to verify fibre placement and virus expression.

### Optogenetics

#### Fibre cannula placement

Male *Tbx21*^ChR2-tdTomato^ mice were placed on either a control diet or a HFD at 8 weeks of age. At 10 weeks of age, an optical fibre cannula (200-μm diameter, 0.48 NA, flat tip, Doric Lenses) was implanted targeting the septal region (AP: 0.6 mm, ML: 0.0 mm, AP: −3 mm) and secured with dental acrylic (Super-Bond C&B, Sun Medical). To verify fibre placement, mice were deeply anaesthetized with ketamine–xylazine after experiments and transcardially perfused.

#### Training and acclimatization to the experimental set-up

Mice were placed in cages connected to the PhenoMaster system (TSE systems) for acclimatization the week before data collection and maintained at 22 °C with ad libitum access to food and water. To reduce stress, mice were previously habituated to the patch cord and last manipulated 2 h before food intake recording.

#### Photostimulation

The MTC projections of *Tbx21*^ChR2-tdTomato^ mice were artificially activated using an LED laser at 473 nm with an output power of 10 mW. Laser stimulation parameters were based on 2P imaging of the dorsal OB (5-ms laser pulse duration generated at 20 Hz for 2-s and 4-s intervals). Laser stimulation was performed for 10 min before the onset of the dark phase (pre-stimulation), for 3 h before the onset of the dark phase (prolonged pre-stimulation), for 10 min after 90 min of the onset of the dark phase (dark-phase stimulation), for 3 h from the onset of the dark phase (prolonged dark-phase stimulation) or for 3 h during the light phase after overnight fasting (refeeding stimulation). Each mouse was its own control and was randomly assigned to the stimulated group (stimulation) or the unstimulated group (no stimulation) during experiments.

#### Food intake recording

Food consumption was recorded using the PhenoMaster system (TSE systems). Food and water intake was measured continuously and simultaneously.

#### Open field test

After 8 weeks of HFD or 4 weeks of control diet, MTC projections were stimulated for 10 min 5 h into the light phase, and the mice were then acclimatized for a period of 60 min. Mice were then placed in the centre of the open area and their exploratory behaviour was recorded for a period of 60 min using the TSE VideoMot 3D Analysis v7.01 software. At 90 min after stimulation, time spent in the central zone, distance travelled, average speed and rearing behaviour were analysed for each mouse for a period of 5 min.

#### Food finding test

Over 3 days, control diet-fed mice were habituated to a highly palatable cookie (Leibniz) and after an overnight fast, MTC projections were stimulated for 10 min during the light phase. Immediately before the experiment, mice were habituated to the cage filled with bedding for 5 min. Ninety minutes after stimulation, mice were placed in the centre of the cages with a cookie hidden in the bedding. Video recordings were used to manually measure the latency to retrieve the hidden cookie.

#### Metabolic hormones

Control diet-fed mice were stimulated for 10 min before dark cycle onset and blood was collected 60 min later during the dark cycle. After clotting at room temperature, samples were centrifuged at 9.6*g* for 20 min at 4 °C. The supernatant was collected and snap-frozen in liquid nitrogen. Samples were then processed using a leptin ELISA kit (Crystal Chem, 90030) and cholecystokinin ELISA Kit (RayBiotech, EIA-CCK-1).

#### Corticosterone ELISA

After 3 weeks on HFD, mice were stimulated for 10 min during the light cycle and blood was collected 90 min later. After clotting at room temperature, samples were centrifuged at 9.6*g* for 20 min at 4 °C. The supernatant was collected and snap-frozen in liquid nitrogen. Samples were then processed using a corticosterone ELISA kit (Crystal Chem, 80556).

### Neuronal activity labelling after odour exposure in freely behaving mice

#### FOS and pS6 staining following odour exposure

Eight-week-old male C57BL/6N mice were used for analysis of neuronal activity by FOS. For activation analysis by phosphorylation of S6 (pS6), C57BL/6N mice were used at 16 weeks of age after 8 weeks on control diet or HFD. One week before the experiments, mice were acclimatized to the presence of a teaball in their home cage and to daily handling to minimize stress and the effects of odours associated with the experimental room, the experiments themselves and the experimenter. Mice were then fasted overnight for 16 h by placing mice into a new cage without food. Mice were then exposed to food odours by placing food pellets in a metal teaball or an empty teaball (control) for 60 or 90 min before transcardial perfusion. The tissue was subsequently processed as described below.

### FOS staining after ZnSO_4_ application

#### Olfactory behaviour and MOE thickness

Adult male C57BL/6N mice (8–14 weeks) were treated with either 10 μl ZnSO_4_ (0.34 M in saline) or 10 μl saline in the right naris. To avoid damage to the oral organs, the mice were immediately placed face down for a few seconds. One hour later, the left naris was injected in the same manner. Four days after ZnSO_4_ application, a food finding test was performed after an overnight fast. Mice were fasted overnight and exposed to food odours before transcardial perfusion. The tissue was subsequently processed as described below.

#### FOS staining

Adult male C57BL/6N mice (10 weeks) were treated with either 50 μl ZnSO_4_ (0.15 M in saline) or 50 μl saline in the right naris. To avoid damage to the oral organs, the solution was applied in small droplets after which the mice were immediately placed face down for a few seconds. Ninety minutes later, the left naris was injected in the same manner. Four days after ZNSO_4_ application, mice were fasted overnight and exposed to food odours before transcardial perfusion. The tissue was subsequently processed as described below.

#### FOS staining after chemogenetic MTC stimulation

Four weeks after AAV9-hSyn-DIO-hM3Dq(Gq)-mCherry injection into the OB of *Tbx21*-Cre mice (8–12 weeks), mice were injected with CNO (1 mg per kg body weight in saline, i.p.; Bio-Techne, 4936) or saline 90 min before transcardial perfusion. The tissue was subsequently processed as described below.

### Histology

#### Tissue processing

For FOS and pS6 induction, mice were exposed to odours for 90 min before euthanasia. For immunohistochemistry, mice were deeply anaesthetized with ketamine–xylazine and transcardially perfused with 0.9% saline followed by phosphate-buffered or borate-buffered 4% paraformaldehyde. Brains were removed, postfixed in 4% paraformaldehyde for 4 h and transferred to 20% sucrose in 0.1 M phosphate-buffered saline (PBS; pH 7.5) overnight. Free-floating brain sections of 30-µm thickness were cut on a cryostat (Leica) and stored at −20 °C in an anti-freeze solution (30% ethylene glycol and 20% glycerol in PBS). Every fourth section was processed for immunohistochemistry as described below.

#### Immunohistochemical staining

Brain tissue analysed for neuronal activity by FOS expression was rinsed with PBS (0.05 M) before sections of 30-µm thickness were washed with glycine (0.3% in PBS). After further washing with PBS followed by incubation with sodium dodecyl sulfate (0.03% in PBS), sections were incubated with normal donkey serum (NDS) or normal goat serum (NGS) to block any non-specific binding sites. The sections were then incubated overnight at room temperature with rabbit anti-FOS (Cell Signaling, 2250S; 1:1,000 dilution in PBS-NDS 3% or 1:1,000 dilution in PBS-NGS 3%), goat anti-tdTomato (Sicgen, AB8181-200; 1:1,000 dilution in PBS-NDS 3%) and rat anti-mCherry (Invitrogen, M11217; 1:1,000 dilution in PBS-NGS 3%). After washing with PBS containing 3% Triton X-100 (PBST), sections were incubated for 2 h at room temperature with donkey anti-rabbit Alexa Fluor 488 (Invitrogen/Thermo Fisher Scientific, A11008; 1:500 dilution in PBS-NDS 3%), donkey anti-goat Alexa Fluor 594 (Invitrogen/Thermo Fisher Scientific, A11058; 1:500 dilution in PBS-NDS 3%), goat anti-rabbit Alexa Fluor 488 (Invitrogen/Thermo Fisher Scientific, A11008; in PBS-NGS 3%) or goat anti-rat Alexa Fluor 594 (Invitrogen/Thermo Fisher Scientific, A11007; in PBS-NGS 3%). For experiments analysing neuronal activity by phosphorylation of ribosomal pS6 (phospho-S6), brains were incubated with NGS and Triton X-100 after washing with PBS. Sections were then incubated with phospho-S6 (pS6; Thermo Fisher Scientific, 4-923G; 1:1,500 dilution in PBST-NGS 2% -bovine serum albumin (BSA) 2%) for 72 h at 4 °C. After washing with PBS, sections were incubated with donkey anti-rabbit Fab fragments (Abcam/Jackson ImmunoResearch, 711-007-003; 1:200 dilution in PBST-BSA 2%) for 1.5 h at room temperature. After further washes in PBS, sections were incubated with goat anti-rabbit Alexa Fluor 594 (Invitrogen/Thermo Fisher Scientific, A11012; 1:500 dilution in PBST-NGS 2%). The sections were then washed in PBS and incubated with NeuroTrace Blue (Thermo Fisher Scientific, N21479; 1:200 dilution in PBST) for 30 min. Brain tissues from fibre photometry experiments, MTC projection tracing and noses from anosmia studies were incubated with NDS or NGS and Triton X-100 after washing with PBS. Sections were then incubated for 72 h at 4 °C with goat anti-OMP (01922291, WAKO Chemicals; 1:1,000 dilution in PBST-NDS 3%), chicken anti-GFP (Abcam/Jackson ImmunoResearch, AB13970; 1:500 dilution in PBST-NGS 3%) or rat anti-mCherry (Invitrogen/Thermo Fisher Scientific, M11217; 1:1,000 dilution in PBS-NGS 3%). After washing with PBS, sections were incubated with donkey anti-goat Alexa Fluor 488 (Abcam/Jackson ImmunoResearch, A11055; 1:500 dilution in PBST-NDS 3%), goat anti-chicken FITC (Abcam/Jackson ImmunoResearch, 103-095-155; 1:500 dilution in PBS-NGS 3%) or goat anti-rat Alexa Fluor 594 (Invitrogen/Thermo Fisher Scientific, A11007; in PBS-NGS 3%). All tissues were finally washed in PBST, mounted on glass slides with Vectashield mounting medium containing DAPI (Biozol, VEC-H-1200) and stored at 4 °C until analysis.

#### Fluorescence in situ hybridization

For morphological analysis of *Slc17a6* expression, brain sections of 20-µm thickness were processed using RNAscope (Advanced Cell Diagnostics (ACD), 323100) and incubated at 40 °C using a humidified chamber and HYbEz oven (ACD), unless otherwise noted. VGlut2 probes (*Slc17a6*; 319171, ACD) were purchased from ACD. Before overnight incubation at 60 °C, slides were thawed and washed with diethylpyrocarbonate-treated Millipore water. Sections were then incubated with hydrogen peroxide for 10 min at room temperature, followed by target retrieval (ACD) at 95 °C for 20 min. After washing in diethylpyrocarbonate water, slides were briefly immersed in ethanol (100%). Slides were then treated with protease III (ACD) for 30 min and incubated with the Slc17a6 probe (channel 2) for 2 h. After washing with wash buffer (ACD), slides were incubated with Amp1 (ACD) for 30 min, Amp2 (ACD) for 30 min and Amp3 (ACD) for 15 min, and the slides were washed with wash buffer (ACD) between amplifications. Slides were then incubated in HRP-C2 solution (ACD) for 15 min, followed by washes in wash buffer (ACD) and incubation with channel 2 probe-associated TSA Plus Cyanine 3 (1:3,000 dilution; BEL760001KT, Perkin Elmer) for 30 min. After washing with wash buffer (ACD), the slides were treated with HRP Blocker Solution (ACD) and wash buffer for 15 min. Finally, the slides were coverslipped with Vectashield mounting medium containing DAPI (Biozol, VEC-H-1200) and stored at 4 °C until analysis.

#### Confocal microscopy

Images were captured using a Leica TCS SP-8-X confocal microscope and a Leica Stellaris microscope equipped with a ×20, ×40 or ×63 objective. To ensure similar imaging conditions between all animals in each experiment, the same microscope set-up was used to capture all images within the same experiments.

#### Quantification

Investigators were blinded at every step of data analysis. Quantification of FOS immunoreactive cells was performed manually at the same rostrocaudal levels of each region using FIJI software. pS6 immunoreactive cells were manually quantified using the integrated density function of FIJI, where the mean intensity is multiplied by the area. The same settings were used to analyse all images within the same experiment to ensure unbiased quantification.

### Ribosome immunoprecipitations (pS6-RiboTrap) after odour exposure

#### Food odour exposure

One week before the experiments, 8-week-old C57BL/6N male mice were acclimatized to the presence of a teaball in their home cage and to daily handling. Mice were then fasted overnight for 16 h by placing mice into a new cage without food. Mice were then exposed to food odours by placing food pellets in a metal teaball or an empty teaball (control) for 60 min.

#### Tissue preparation and ribosome immunoprecipitation

Phosphorylated ribosome pulldown for identification of neuronal activation was adapted from previous protocols^100^. One hour after exposure to food odours, mice were euthanized by decapitation. The septal region was rapidly dissected using a brain block on ice in buffer containing 1× HBSS (Gibco, with Ca^2+^ and Mg^2+^), 2.5 mM HEPES (pH 7.4), 35 mM glucose, 100 μg ml^−1^ cycloheximide, 5 mM sodium fluoride, 1 mM sodium orthovanadate, 1 mM sodium pyrophosphate and 1 mM beta-glycerophosphate. Five septal regions were pooled for homogenization ten times at 250 rpm and nine times at 750 rpm on a rotating glass/Teflon Potter homogenizer (Potter S, Braun) at 4 °C in 1 ml buffer containing 150 mM KCl, 5 mM MgCl^2^, 10 mM HEPES, 100 nM calyculin A, 2 mM dithiothreitol, 100 U RNasin (Promega), 100 μg cycloheximide, one tablet of Complete mini EDTA-free protease inhibitor cocktail/7 ml and one tablet of PhosSTOP/5 ml. Samples were spun in 1.5-ml LoBind tubes (Eppendorf) and centrifuged at 2,000*g* for 10 min at 4 °C. Next, 90 μl 10% NP-40 (vol/vol) and 90 μl 300 mM DHPC (Avanti Polar Lipids) were added to the supernatant, which was centrifuged again at 17,000*g* for 10 min at 4 °C. The supernatant was transferred to a new LoBind tube and mixed with 20 μl of pS6 antibody (Cell Signaling 2215). Protein A Dynabeads (Invitrogen, 100 μl per sample) were washed three times with 900 μl buffer containing 150 mM KCl, 5 mM MgCl^2^, 10 mM HEPES, 0.05% BSA (wt/vol) and 1% NP-40. After antibody binding, the mixture was pipetted onto the washed and mixed beads and incubated for 1 h at 4 °C on a rotator. After incubation, the ribosome-bound beads were washed four times with 700 μl buffer containing 350 mM KCl, 5 mM MgCl^2^, 10 mM HEPES, 1% NP-40, 2 mM dithiothreitol, 100 U ml^−1^ recombinant RNasin (Promega), 100 μg ml^−1^ cycloheximide, 5 mM sodium fluoride, 1 mM sodium orthovanadate, 1 mM sodium pyrophosphate and 1 mM beta-glycerophosphate. During the final wash, the beads were placed on the magnet and allowed to equilibrate to room temperature. After removing the supernatant, RNA was eluted by mixing the beads with 350 μl RLT (from Qiagen RNeasy kit). The eluted RNA was purified using the RNeasy Micro Kit (Qiagen). Chemicals were purchased from Sigma unless otherwise stated. The quality of the purified RNA was confirmed using an Agilent 2100 bioanalyzer with an Agilent RNA 6000 Pico kit.

#### Gene expression analysis

cDNA was prepared by reverse transcription (High-capacity cDNA Reverse Transcription Kit, Applied Biosciences) and amplified using TaqMan Universal PCR Master Mix (4368814; Applied Biosystems). All samples were treated with DNase (79254; Qiagen). Relative expression of target mRNAs was corrected for total RNA content (GAPDH; Mm 99999915_g1; Thermo Fisher Scientific). The following inventoried TaqMan probes were used: *Calb2* (Mm00801461_m1; Thermo Fisher Scientific), *Nts* (Mm00481140_m1; Thermo Fisher Scientific), *Slc17a6* (Mm00499876_m1; Thermo Fisher Scientific), *Slc32a1* (Mm00494138_m1; Thermo Fisher Scientific) and *Sst* (Mm00436671_m1; Thermo Fisher Scientific). Calculations were performed using a comparative method (2^−ΔΔCT^). Quantitative PCR was performed on an AB-QuantStudio7Flex (Applied Biosystems). Values reported are the ratio of pulldown expression to input expression, normalized to the average of the unexposed group for each gene.

### PET imaging

#### PET imaging procedure

PET imaging was performed with an Inveon preclinical PET/computed tomography system (Siemens) as previously described^101^.

#### Olfactory exposure during PET imaging

One week before the experiments, 8-week-old C57BL/6N male mice were acclimatized to the experimental rooms and daily handling to minimize the effects of odours associated with the experimental room and the experimenter. Food odours were introduced into the air stream of each mouse’s anaesthesia mask immediately before the start of the measurements.

#### Schematic visualization of ^18^FDG-PET brain activity

For schematic visualization of regional activity induced by food odour exposure, the magnitude of *P* values was used to create a spatial map indicating food odour-induced activity through orange circles. For colour-grading indication of regional activity, the voxel-based analyses of the ratio of tissue and plasma glucose concentrations (CE/CP) based on ^18^FDG kinetics in brains of fasted C57BL/6N mice exposed or not to food odours were used. Here, the average CE/CP of all regions was utilized to normalize each individual regional CE/CP in response to food odours or no odours.

### In vivo 2P imaging

In vivo 2P imaging has been performed as previously described^102,103^.

### Statistical analysis

Statistical analysis was performed using GraphPad Prism 10. Data distribution was assumed to be normal, but this was not formally tested. Datasets comparing experiments in the same animal subjected to two different treatments were analysed for statistical significance using a paired two-tailed Student’s *t*-test. Datasets with only two independent groups were analysed for statistical significance using an unpaired two-tailed Student’s *t*-test. Datasets with more than two groups were analysed using a one-way ANOVA followed by a multiple-comparison test. Datasets with two independent factors were analysed using a two-way ANOVA followed by Sidak’s multiple-comparison test. Alpha was defined as 0.05. No statistical methods were used to predetermine sample sizes, although group sizes used were similar to those commonly applied in mouse studies.

### Reporting summary

Further information on research design is available in the Nature Portfolio Reporting Summary linked to this article.

## Supplementary information

Reporting Summary

## Source data

Source Data Fig. 1 Raw data, *n* numbers and statistical summary.

Source Data Fig. 2 Raw data, *n* numbers and statistical summary.

Source Data Fig. 3 Raw data, *n* numbers and statistical summary.

Source Data Fig. 4 Raw data, *n* numbers and statistical summary.

Source Data Fig. 5 Raw data, *n* numbers and statistical summary.

Source Data Fig. 7 Raw data.

Source Data Fig. 7 *n* numbers and statistical summary.

Source Data Fig. 8 Raw data, *n* numbers and statistical summary.

Source Data Extended Data Fig. 1 Raw data, *n* numbers and statistical summary.

Source Data Extended Data Fig. 2 Raw data, *n* numbers and statistical summary.

Source Data Extended Data Fig. 3 Raw data, *n* numbers and statistical summary.

Source Data Extended Data Fig. 4 Raw data, *n* numbers and statistical summary.

Source Data Extended Data Fig. 5 Raw data, *n* numbers and statistical summary.

Source Data Extended Data Fig. 6 Raw data, *n* numbers and statistical summary.

Source Data Extended Data Fig. 7 Raw data, *n* numbers and statistical summary.

Source Data Extended Data Fig. 8 Raw data, *n* numbers and statistical summary.

Source Data Extended Data Fig. 9 Raw data, *n* numbers and statistical summary.

Source Data Extended Data Fig. 10 Raw data, *n* numbers and statistical summary.

## Figures and Tables

**Extended Data Table. 10:**
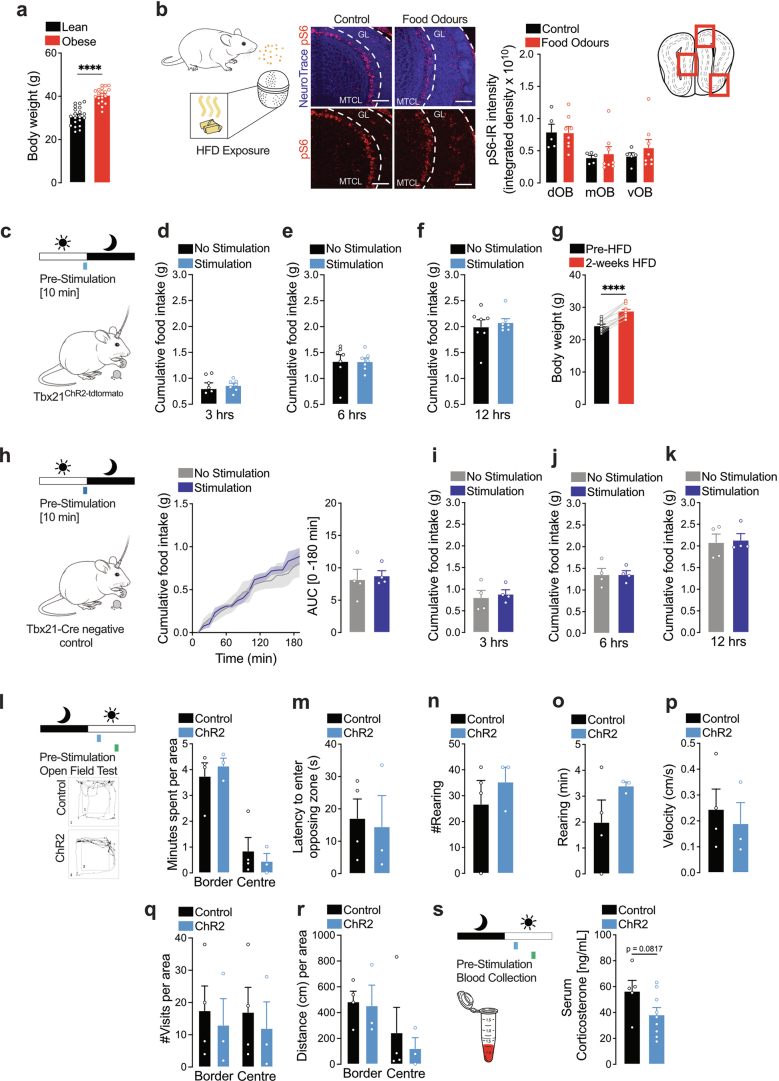
Optogenetic stimulation of OB→MS projections did not induce a change in feeding behaviour in diet-obese mice. **a)** Bodyweight of CD-fed (lean) and HFD-fed (obese) mice when performing the food finding test [two-tailed Student’s t-test; n = 20 mice]. **b)** Representative images of the dorsal olfactory bulb (dOB) and quantification comparison of food odour-induced (HFD) pS6 immunoreactivity (pS6, green; Neurotrace, blue) of the dOB, medial olfactory bulb (mOB) and ventral olfactory bulb (vOB) [Student’s t-test; n = 5 mice for control, n = 8 mice for food odours]. Scale bar: 100 µm. **c)** Cumulative food intake of obese Tbx21^ChR2-tdTomato^ mice following a 10-minute stimulation prior to the dark cycle after **d)** three hours, **e)** six hours and **f)** twelve hours [two-tailed Student’s t-test; n = 7 mice per group]. **g)** Bodyweight of Tbx21^ChR2-tdTomato^ mice before and after two weeks on HFD. **h)** Cumulative food intake and AUC of Tbx21-Cre negative control obese animals following a 10-minute pre-stimulation before the dark cycle [time curve: two-way ANOVA, Sidak post-hoc; n = 4 mice per group] [AUC: two-tailed Student’s t-test; n = 4 mice per group]. Cumulative food intake of Tbx21-Cre negative control animals after **i)** three hours, **j)** after six hours and **k)** after twelve hours following a 10-minute stimulation before dark cycle [two-tailed Student’s t-test, n = 4 mice per group]. **l-r)** Open field test 60 minutes after a 10-minute stimulation of obese Tbx21^ChR2-tdTomato^ (ChR2) and Tbx21-Cre negative (control) mice during the light cycle depicted as **l)** minutes spent per area [two-way ANOVA, Tukey post-hoc; n = 3 control mice, n = 4 ChR2 mice], **m)** latency to enter the opposing area [two-tailed Student’s t-test; n = 3 control mice, n = 4 ChR2 mice], **n)** number and **o)** minutes of rearing [two-tailed Student’s t-test; n = 3 control mice, n = 4 ChR2 mice], **p)** velocity [two-tailed Student’s t-test; n = 3 control mice, n = 4 ChR2 mice], **q)** number of visits and **r)** distance travelled per area [two-way ANOVA, Tukey post-hoc; n = 3 control mice, n = 4 ChR2 mice]. **s)** Corticosterone level in blood serum 60 minutes after a 10-minute stimulation of obese Tbx21^ChR-tdTomato^ (ChR2) and Tbx21-Cre negative (control) mice during the light cycle [two-tailed Student’s t-test; n = 5 control mice, n = 9 ChR2 mice]. Data are represented as mean ± SEM. *p ≤ 0.05, **p ≤ 0.01, ***p ≤ 0.001 and, ****p ≤ 0.0001. dOB, dorsal olfactory bulb; mOB, medial olfactory bulb; vOB, ventral olfactory bulb; GL, glomerular layer; MTCL, mitral and tufted cell layer. Eppendorf image in **s** and mouse images in **b**, **c** and **h** adapted from SciDraw under a Creative Commons license CC BY 4.0. Source data

## Data Availability

Surgery co-ordinates were based on and PET images were co-registered to the Paxinos brain atlas^93^. Illustrations of sniffing mice were adapted from BioRender.com (Figs. 3a,h, 4d–g and 5a–d and Extended Data Figs. 2b, 3a,b, 4a,e,g, 5a–c, 6a and 7a,b). Illustration of mice (Figs. 2c,d, 3d–f, 6a–i, 7a,e,h–l and 8a–c and Extended Data Figs. 2e, 3c, 6c, 7e,f, 8d,j, 9a,e and 10b,c,h) and Eppendorf tubes (Fig. 3a and Extended Data Figs. 9q and 10s) were sourced from https://scidraw.io/ and adapted from Tyler, E., & Kravitz, L. (2020) and Losch De Oliveira, D. (2020), respectively. Source data are provided with this paper.

## Code availability

Code generated for fibre photometry calcium recordings is available on request. Code generated for 2P imaging has been previously described^102,103^.
