# Supplementary material for: A food-sensitive olfactory circuit drives anticipatory satiety
Source: Nat Metab. 2025 Jun 11;7(6):1246–65. doi: 10.1038/s42255-025-01301-1 (PMC12198014; doi:10.1038/s42255-025-01301-1)
Supplement: Supplementary file 1 — Reporting Summary [file 42255_2025_1301_MOESM1_ESM.pdf]

nature portfolio

Corresponding author(s): Dr. Sophie Steculorum
Last updated by author(s): 13/03/2025

Reporting Summary

Nature Portfolio wishes to improve the reproducibility of the work that we publish. This form provides structure for consistency and transparency in reporting. For further information on Nature Portfolio policies, see our Editorial Policies and the Editorial Policy Checklist.

Statistics

For all statistical analyses, confirm that the following items are present in the figure legend, table legend, main text, or Methods section.

n/a	Confirmed	
☐	☒	The exact sample size (*n*) for each experimental group/condition, given as a discrete number and unit of measurement
☐	☒	A statement on whether measurements were taken from distinct samples or whether the same sample was measured repeatedly
☐	☒	The statistical test(s) used AND whether they are one- or two-sided<br>*Only common tests should be described solely by name; describe more complex techniques in the Methods section.*
☒	☐	A description of all covariates tested
☐	☒	A description of any assumptions or corrections, such as tests of normality and adjustment for multiple comparisons
☐	☒	A full description of the statistical parameters including central tendency (e.g. means) or other basic estimates (e.g. regression coefficient) AND variation (e.g. standard deviation) or associated estimates of uncertainty (e.g. confidence intervals)
☐	☒	For null hypothesis testing, the test statistic (e.g. *F*, *t*, *r*) with confidence intervals, effect sizes, degrees of freedom and *P* value noted<br>*Give P values as exact values whenever suitable.*
☒	☐	For Bayesian analysis, information on the choice of priors and Markov chain Monte Carlo settings
☒	☐	For hierarchical and complex designs, identification of the appropriate level for tests and full reporting of outcomes
☒	☐	Estimates of effect sizes (e.g. Cohen's *d*, Pearson's *r*), indicating how they were calculated

Our web collection on statistics for biologists contains articles on many of the points above.

Software and code

Policy information about availability of computer code

Data collection	o For ELISA, the optical density was determined using a FilterMax F5 Mulit-Mode microplate reader and SoftMax Pro6.3 software (V.6.3.0.1, Molecular Devices).<br>o PET imaging was performed using an Inveon preclinical PET/CT system (Siemens).<br>o Photometry recordings were performed using Synapse software (V.92, Tucker-Davis Technologies, TDT, USA)<br>o Tissue was imaged on a Zeiss Imager M2 microscope and Axio software (V 4.2, Carl Zeiss) or a Leica STELLARIS (Leica Microsystems)<br>o Quantitative PCR was performed on an AB-QuantStudio7Flex (V1.7.1, Applied Biosystems).<br>o Mouse behavior during an open field test were recorded using a TSE VideoMOt 3D Analysis software (V7.01, TSE systems, Germany).<br>o Volatiles measurements were performed using a 200C: miniPID Fast Response Olfaction Sensor, Aurora Scientific.
Data analysis	o Data Analysis was performed using Graphpad Prism (V.10; Graphpad Software Inc, La Jolla, CA, USA) unless otherwise stated.<br>o All fiber photometry data analyses were preformed using a custom Matlab script (V.R2021a; available upon request)<br>o Analysis of PET Imaging was performed using the Vinci software (V.5.060).<br>o Results of quantitative PCR experiments were calculated using Microsoft Excel (V.16.42).

For manuscripts utilizing custom algorithms or software that are central to the research but not yet described in published literature, software must be made available to editors and reviewers. We strongly encourage code deposition in a community repository (e.g. GitHub). See the Nature Portfolio guidelines for submitting code & software for further information.

Data

Policy information about availability of data

All manuscripts must include a data availability statement. This statement should provide the following information, where applicable:

- Accession codes, unique identifiers, or web links for publicly available datasets
- A description of any restrictions on data availability
- For clinical datasets or third party data, please ensure that the statement adheres to our policy

o Raw data from experiments, summary statistical analysis including exact p-values are provided in source data files.
o Surgery coordinates were based on and PET images were co-registered to the Paxinos Brain Atlas (Paxinos et al., 2001).

Human research participants

Policy information about studies involving human research participants and Sex and Gender in Research.

Reporting on sex and gender	N/A
Population characteristics	N/A
Recruitment	N/A
Ethics oversight	N/A

Note that full information on the approval of the study protocol must also be provided in the manuscript.

Field-specific reporting

Please select the one below that is the best fit for your research. If you are not sure, read the appropriate sections before making your selection.

☒ Life sciences ☐ Behavioural & social sciences ☐ Ecological, evolutionary & environmental sciences

For a reference copy of the document with all sections, see nature.com/documents/nr-reporting-summary-flat.pdf

Life sciences study design

All studies must disclose on these points even when the disclosure is negative.

Sample size	No statistical methods were used to pre-determined sample sizes although group sizes used were similar to those commonly applied in mouse studies.
Data exclusions	For the photometry analyses, mice showing no viable fluorescent signals were excluded. All exclusion criterion were pre-established.
Replication	o Every mouse represents a replicate and the number of replicates is mentioned for each experiment. Experiments were performed in cohorts generated at different time points.<br>o For food intake and fibre photometry calcium recordings, littermates from at least 3 cohorts were used. No significant differences were observed.<br>o For AAV- and rabies based tracing and RNAscope studies, we aimed for a minimum of 2-3 mice per condition. All attempts were successful.
Randomization	Mice have been assigned randomly to each groups and experiments.
Blinding	Investigators were blinded during experiments and analysis when applicable.

Reporting for specific materials, systems and methods

We require information from authors about some types of materials, experimental systems and methods used in many studies. Here, indicate whether each material, system or method listed is relevant to your study. If you are not sure if a list item applies to your research, read the appropriate section before selecting a response.

Materials & experimental systems

n/a	Involved in the study
☐	☒ Antibodies
☒	☐ Eukaryotic cell lines
☒	☐ Palaeontology and archaeology
☐	☒ Animals and other organisms
☒	☐ Clinical data
☒	☐ Dual use research of concern

Methods

n/a	Involved in the study
☒	☐ ChIP-seq
☒	☐ Flow cytometry
☒	☐ MRI-based neuroimaging

Antibodies

Antibodies used

o rabbit anti-FOS (Cell Signaling, 2250S; 1/1000)
o goat anti-tdTomato (Sicgen antibodies; AB8181-200; 1/1000)
o rat anti-mCherry (Invitrogen, M11217; 1/1000)
o goat anti-OMP (01922291, WAKO Chemicals; 1/1000)
o chicken anti-GFP (Abcam / Jackson Immunoresearch, AB13970; 1/500)
o Anti-phospho-S6 ribosomal protein (Ser 244/247) (Thermo Fisher Scientific, 4-923G; 1/1500)
o Anti-phospho-S6 ribosomal protein (Ser 240/244) (Cell Signaling, 2215; approx. 1/60)
o NeuroTrace Blue (Thermo Fisher Scientific, N21479; 1/200)
o donkey anti-rabbit Alexa Fluor 488 (Invitrogen / Thermo Fisher Scientific, A11008; 1/500)
o donkey anti-goat Alexa Fluor 594 (Invitrogen / Thermo Fisher Scientific, A11058; 1/500)
o donkey anti-goat Alexa Fluor 488 (Invitrogen / Thermo Fisher Scientific, A11055; 1/500)
o goat anti-rabbit Alexa Fluor 488 (Invitrogen / Thermo Fisher Scientific, A11008; 1/500)
o goat anti-rabbit Alexa Fluor 594 (Invitrogen / Thermo Fisher Scientific, A11012; 1/500)
o goat anti-rat Alexa Fluor 594 (Invitrogen / Thermo Fisher Scientific, A11007; 1/500)
o goat anti-chicken FITC (Abcam / Jackson Immunoresearch, 103-095-155; 1/500)

Validation

Antibodies were validate by the manufacturer company:

o rabbit anti-FOS (Cell Signaling), cited by >1000 publications. https://www.cellsignal.com/products/primary-antibodies/c-fos-9f6-rabbit-mab/2250?srsltid=AfmBOop-oyejyEx9Q986tWAdP7Wqse0F-yEvsBwc7zaag-zdegdSoii3
o goat anti-tdTomato (Sicgen antibodies), cited by >100 publications. https://store.sicgen.pt/catalog/product/AB8181
o rat anti-mCherry (Invitrogen), cited in 431 publications. https://www.thermofisher.com/antibody/product/mCherry-Antibody-clone-16D7-Monoclonal/M11217
o goat anti-OMP (WAKO Chemicals) cited 10 time in webpage. https://labchem-wako.fujifilm.com/europe/product/detail/W01W0101-2229.html
o chicken anti-GFP (Abcam / Jackson Immunoresearch) cited in 5189 publications. https://www.abcam.com/en-us/products/primary-antibodies/gfp-antibody-ab13970?srsltid=AfmBOorYdYMEQeeW87e52u3sijHHihn_q-99t1A5g9nelvwQD88K3rFx
o Anti-phospho-S6 ribosomal protein (Ser 244/247) (Thermo Fisher Scientific) cited in >20 publications. https://www.thermofisher.com/antibody/product/Phospho-S6-Ser244-Ser247-Antibody-Polyclonal/44-923G
o Anti-phospho-S6 ribosomal protein (Ser 240/244) (Cell Signaling) cited in >900 publications. https://www.cellsignal.com/products/primary-antibodies/phospho-s6-ribosomal-protein-ser240-244-antibody/2215?srsltid=AfmBOoom8HeoPSmU-AKutq96OjsIpOOSZiwqYOMzLuwdhk-GbgFYJWWI
o NeuroTrace Blue (Thermo Fisher Scientific, N21479) cited in >20 publications. https://www.thermofisher.com/order/catalog/product/N21479/tabs?defaultTab=4
o donkey anti-rabbit Alexa Fluor 488 (Invitrogen / Thermo Fisher Scientific, A11008) cited in >10000 publications. https://www.thermofisher.com/antibody/product/Goat-anti-Rabbit-IgG-H-L-Cross-Adsorbed-Secondary-Antibody-Polyclonal/A-11008
o donkey anti-goat Alexa Fluor 594 (Invitrogen / Thermo Fisher Scientific, A11058) cited in >1000 publications. https://www.thermofisher.com/antibody/product/Donkey-anti-Goat-IgG-H-L-Cross-Adsorbed-Secondary-Antibody-Polyclonal/A-11058
o donkey anti-goat Alexa Fluor 488 (Invitrogen / Thermo Fisher Scientific, A11055) cited in >3000 publications. https://www.thermofisher.com/antibody/product/Donkey-anti-Goat-IgG-H-L-Cross-Adsorbed-Secondary-Antibody-Polyclonal/A-11055
o goat anti-rabbit Alexa Fluor 488 (Invitrogen / Thermo Fisher Scientific, A11008) cited in > 10.000 publications. https://www.thermofisher.com/antibody/product/Goat-anti-Rabbit-IgG-H-L-Cross-Adsorbed-Secondary-Antibody-Polyclonal/A-11008
o goat anti-rabbit Alexa Fluor 594 (Thermo Fisher Scientific) cited in >3.000 publications. https://www.thermofisher.com/antibody/product/Goat-anti-Rabbit-IgG-H-L-Cross-Adsorbed-Secondary-Antibody-Polyclonal/A-11012
o goat anti-rat Alexa Fluor 594 (Invitrogen / Thermo Fisher Scientific) cited in >900 publications. https://www.thermofisher.com/antibody/product/Goat-anti-Rat-IgG-H-L-Cross-Adsorbed-Secondary-Antibody-Polyclonal/A-11007
o goat anti-chicken FITC (Abcam / Jackson Immunoresearch) https://www.jacksonimmuno.com/catalog/products/103-005-155

Animals and other research organisms

Policy information about studies involving animals; ARRIVE guidelines recommended for reporting animal research, and Sex and Gender in Research

Laboratory animals

o Adult male mice were housed in individually ventilated cages at 22-24°C with a 12-hour light/12-hour dark cycle. Animals had ad libitum access to water and food at all times unless otherwise stated. Unless otherwise stated, all experiments were performed on adult male mice aged 8-16 weeks.

o C57BL/6N mice were obtained from Charles River (Germany) and allowed to acclimatize to the animal facility for at least a week prior to the experiment. Tbx21-Cre (JAX stock #024507), VGLUT2-IRES-Cre (JAX stock #016963), Rosa26-CAG-LSL-Synaptophysin-tdTomato-WPRE (JAX stock #012570), and R26-LSL-ChR2(H134R)-TdTomato-WPRE mice (JAX stock #012567) were originally obtained from The Jackson Laboratory and colonies were maintained at the Max Planck Institute for Metabolic Research, Cologne, Germany. The mice used in the experiments were obtained from in-house breeding. Tbx21Synaptophysin-tdtomato (tg/wt; fl/fl or fl/wt) mice were obtained by breeding Tbx21-Cre tg/wt and R26-Synaptophysin-tdTomato fl/fl mice. Tbx21ChR2-tdtomato (tg/wt; fl/fl) mice were obtained by breeding Tbx21-Cre tg/wt and R26-ChR2-tdtomato fl/fl mice. For 2-photon calcium imaging, Tbx21GCaMP6f mice were obtained and maintained at the Francis Crick Institute by breeding Tbx21-Cre and Ai95(RCL-GCaMP6f)-D (GCaMP6f; JAX, stock #024105) mice.

Wild animals	This study did not use wild animals.
Reporting on sex	All experiments reported in the manuscript have been performed in male mice.
Field-collected samples	We did not use field-collected samples in this study.
Ethics oversight	Permission to maintain and breed mice was issued by the Department for Environment and Consumer Protection - Veterinary Section, Cologne, North Rhine-Westphalia, Germany.

Note that full information on the approval of the study protocol must also be provided in the manuscript.
