# Supplementary figures and images for: A food-sensitive olfactory circuit drives anticipatory satiety
Source: Nat Metab. 2025 Jun 11;7(6):1246–65. doi: 10.1038/s42255-025-01301-1 (PMC12198014; doi:10.1038/s42255-025-01301-1)

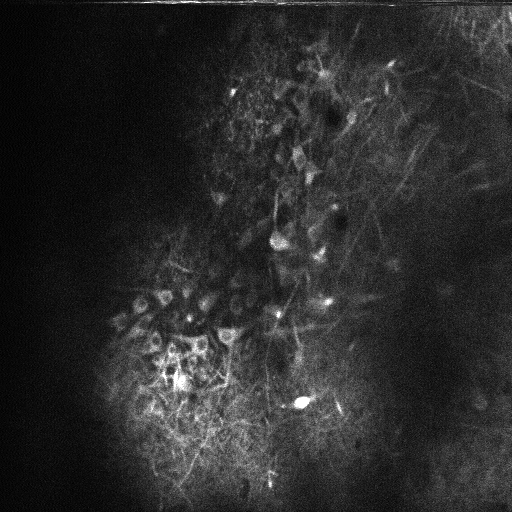

Supplement: Supplementary file 8 — n numbers and statistical summary. [file 42255_2025_1301_MOESM8_ESM.zip › Main Figure/Figure 7b.tif]

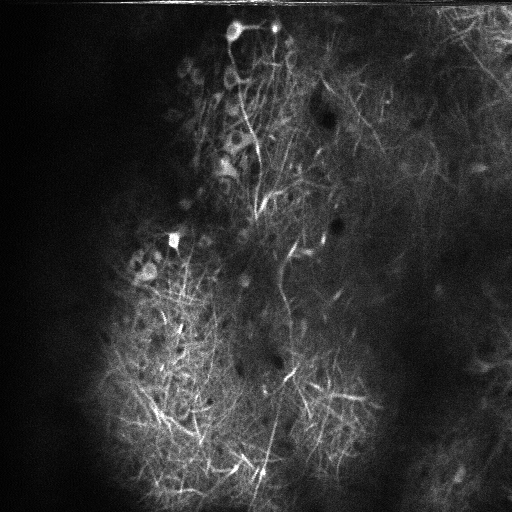

Supplement: Supplementary file 17 — Raw data, n numbers and statistical summary. [file 42255_2025_1301_MOESM17_ESM.zip › Supplementary figure/FigureS8dii.tif]

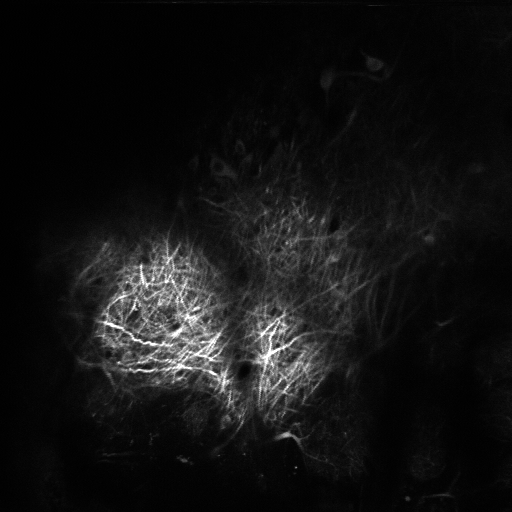

Supplement: Supplementary file 17 — Raw data, n numbers and statistical summary. [file 42255_2025_1301_MOESM17_ESM.zip › Supplementary figure/FigureS8h.tif]

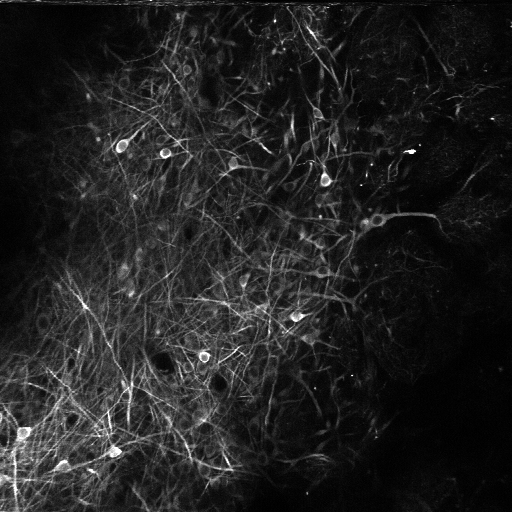

Supplement: Supplementary file 17 — Raw data, n numbers and statistical summary. [file 42255_2025_1301_MOESM17_ESM.zip › Supplementary figure/FigureS8ji.tif]

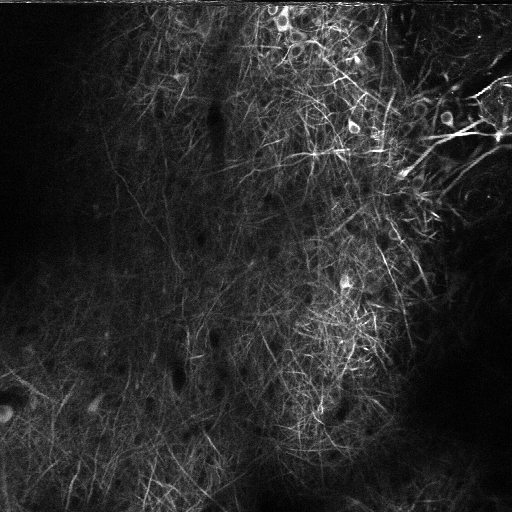

Supplement: Supplementary file 17 — Raw data, n numbers and statistical summary. [file 42255_2025_1301_MOESM17_ESM.zip › Supplementary figure/FigureS8n.tif]

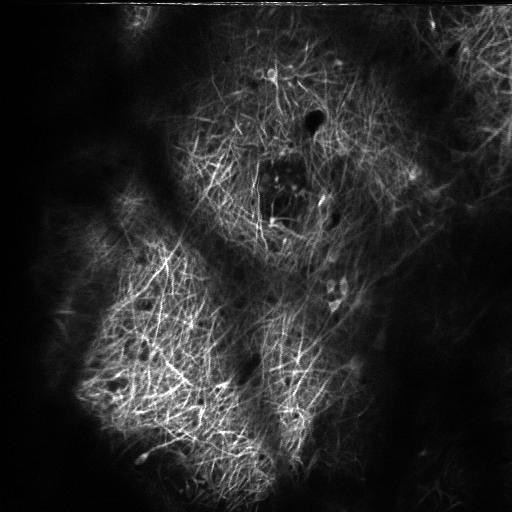

Supplement: Supplementary file 17 — Raw data, n numbers and statistical summary. [file 42255_2025_1301_MOESM17_ESM.zip › Supplementary figure/FigureS8di.tif]

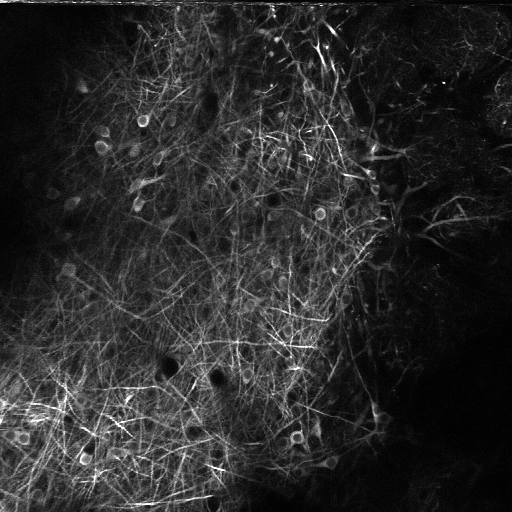

Supplement: Supplementary file 17 — Raw data, n numbers and statistical summary. [file 42255_2025_1301_MOESM17_ESM.zip › Supplementary figure/FigureS8jii.tif]
